# CRISPR-Cas9 enables conditional mutagenesis of challenging loci

**DOI:** 10.1038/srep32326

**Published:** 2016-09-01

**Authors:** Joel A. Schick, Claudia Seisenberger, Joachim Beig, Antje Bürger, Vivek Iyer, Viola Maier, Sajith Perera, Barry Rosen, William C. Skarnes, Wolfgang Wurst

**Affiliations:** 1 Institute of Developmental Genetics, Helmholtz Zentrum München, Ingolstädter Landstraße 1, 85764 Neuherberg/Munich, Germany; 2 Wellcome Trust Sanger Institute, Wellcome Trust Genome Campus, Hinxton, Cambridge, UK; 3 AstraZeneca Darwin Building (Unit 310), Cambridge Science Park, Milton Road, Cambridge, CB4 0WG, United Kingdom; 4 Technische Universität München-Weihenstephan, c/o Helmholtz Zentrum München, Ingolstädter Landstr. 1, 85764 Neuherberg/Munich, Germany; 5 German Center for Neurodegenerative Diseases (DZNE), Site Munich, Feodor-Lynen-Strasse 17, 81377 Munich, Germany; 6 Munich Cluster for Systems Neurology (SyNergy), Adolf-Butenandt-Institut Ludwig-Maximilians-Universität München, Feodor-Lynen-Strasse 17, 81377 Munich, Germany

## Abstract

The International Knockout Mouse Consortium (IKMC) has produced a genome-wide collection of 15,000 isogenic targeting vectors for conditional mutagenesis in C57BL/6N mice. Although most of the vectors have been used successfully in murine embryonic stem (ES) cells, there remain a set of nearly two thousand genes that have failed to target even after several attempts. Recent attention has turned to the use of new genome editing technology for the generation of mutant alleles in mice. Here, we demonstrate how Cas9-assisted targeting can be combined with the IKMC targeting vector resource to generate conditional alleles in genes that have previously eluded targeting using conventional methods.

The production of conditional mutations in mice offers substantial advantages in carrying out cell type- and temporal-specific studies. In addition to circumventing early lethality for some genes, conditional mutations allow detailed investigation into gene function using precise genetic tools in a tissue of interest. Thus, the overarching goal of the IKMC, the European Conditional Mouse Mutagenesis Program (EUCOMM and EUCOMMTOOLS) and the NIH-funded Knockout Mouse Project (KOMP) has been to generate conditional alleles in every protein-coding gene in C57BL/6N mice and to archive and distribute these strains to the scientific community. The EUCOMMTOOLs program has also expanded the collection of transgenic Cre strains available in the C57BL/6N genetic background to facilitate conditional loss-off-function studies in adult tissues^1^,^2^,^3^.

IKMC vectors are typically deployed in a so-called “knockout first, conditional ready” configuration for initial targeting^4^,^5^. The allele contains a lacZ reporter cassette upstream of a LoxP-flanked critical exon. The critical exon is carefully chosen such that subsequent deletion of the exon with Cre recombinase results in a frame-shift mutation in all protein-coding transcripts and a corresponding null mutation^6^. The presence of site-specific recombination sites permits the generation of a conditional allele with Flp recombinase or a lacZ-tagged null allele with Cre recombinase. To identify correctly targeted events following electroporation, genotyping primers are computationally designed to identify positive clones by long-range PCR and sequencing. Nevertheless, 1,859 genes representing 13% of high-throughput targeting experiments in ES cells failed to produce correctly targeted clones, even after repeated attempts (See Supplemental Table 1). The reasons for these failures are not entirely clear, but possible explanations include inefficient recombination of the vector (targeting failure) or an inability to amplify the target region by PCR (genotyping failure). Targeting failures are notoriously difficult to characterize but may be due to ectopic insertions where double-strand breaks occur, tandem vector insertions, or erroneous recombination on one side, for example. In some instances, non-isogenicity between the targeting vector (derived from C57BL/6J BACs) and the target gene (in ES cells derived from the C57BL/6N substrain) may also account for low targeting efficiency^7^. Furthermore, since strand breakage is thought to occur prior to homologous recombination^8^,^9^, well-protected or heterochromatic DNA may preclude breaks in these areas at the same time the targeting vector is in proximity for recombination.

CRISPR technology holds the promise to overcome these barriers as double-strand breaks at the intended genomic site activate DNA repair machinery and allow for highly efficient recombination with targeting vectors containing minimal homology to the endogenous locus^10^,^11^. Already CRISPR-assisted targeting in stem cells has been successfully employed and there are a number of reports displaying its effectiveness^12^,^13^,^14^. Because of its high efficiency, biallelic targeting and multiplexed targeting has been possible^15^,^16^, setting the stage for substantial improvements in mutant production. However, CRISPR-assisted homologous recombination has not yet been examined on a large scale in mouse embryonic stem cells. We therefore chose to investigate if CRISPR could enable targeting at a large number of genomic locations that were previously inaccessible by conventional targeting.

## Results

**Figure 1:**
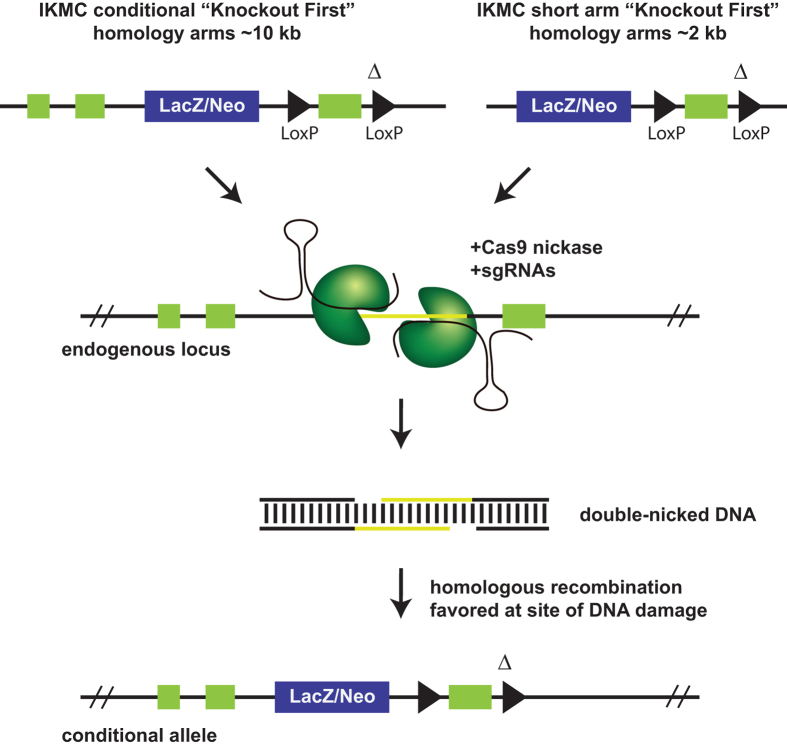
CRISPR-assisted conditional mutagenesis strategy. Single guide RNAs (sgRNAs) are targeted to a small deleted region near the LoxP site in the genomic intron which is absent in IKMC conditional targeting vectors (Δ), resulting in cleavage of the genomic locus but not the targeting vector. Using the dual-nickase strategy, Cas9[D10A] generates a single strand break on both antiparallel strands, triggering repair by homologous recombination. Traditional IKMC long arm homology conditional vectors as well as short arm vectors derived from IKMC intermediate vectors are amenable for assisted mutagenesis. The final allele is indistinguishable from conventional targeted conditional alleles from IKMC with a floxed critical exon that can be removed with Cre recombinase and results in a null allele. The yellow region is deleted in IKMC targeting vectors (Δ). Mock exons are shown as green boxes.

CRISPR offers substantial advantages for mutagenesis as the required elements Cas9 and sgRNA can be delivered transiently along with a conditional targeting vector. A key aspect of IKMC conditional vectors is the presence of a small intronic deletion (average 63 bp) located downstream of the critical exon at the 3′ LoxP site which have been introduced during the automated recombineering oligonucleotide design process^6^. This deletion is predicted to have no effect on gene function (although this must be confirmed on an individual gene basis) and is particularly advantageous as it enables the design of single guide RNAs (sgRNAs) that target genomic DNA at that location without cleaving the targeting vector (Fig. 1). This both ensures vector circularity prior to recombination and hinders spontaneous integration into the genome at ectopic sites.

We chose to employ the dual-nickase Cas9 mutagenesis strategy^17^,^18^ to promote homologous recombination and to minimize off-target damage at highly-related sites. We selected conventional long arm (~5 kb) IKMC targeting vectors for a set of 75 genes that had previously failed to produce targeted clones at least once (37), twice (18) and more (20), with an mean of 1.97 attempts per gene and computationally designed paired sgRNAs where at least one of the sgRNAs targets is directed to the small intronic deletion^19^ (see Methods). Uncut, circular conditional targeting vectors were co-delivered with plasmids expressing Cas9[D10A]nickase^20^ and paired sgRNAs. In light of published reports of efficient targeting with shorter homology arms with CRISPR and other site-specific nucleases^21^,^22^,^23^ we selected another set of 177 failed IKMC projects (mean failed attempts = 1.79) and generated corresponding short arm (~1 kb) vectors by linear-linear gap repair recombineering of IKMC intermediate vectors followed by a two-way Gateway reaction^6^,^24^ (Supplemental Fig. 1; see also Methods). Following electroporation into ES cells, genotyping was carried out on individual colonies using genomic PCR and Sanger sequencing of amplified products (Supplemental File 1).

Our results demonstrate that more than half of the genes which previously failed conventional targeting can be recovered by Cas9[D10A ]-assisted targeting (Table 1). For the long-arm vectors, 35 of 75 genes (47%) were successfully targeted, whereas a higher fraction 111 of 179 (62%) of genes were targeted with short arm vectors. As expected, the efficiency of homologous recombination also increased dramatically in the presence of Cas9 nickase compared to conventional targeting (Supplemental Fig. 2, 34% vs. 18%, *P* < 0.0001, two-tailed t-test). Contrary to the rules governing conventional targeting^25^, the length of genomic homology (5 kb vs. 1 kb arms) does not appear to exert a strong effect on the efficiency of Cas9[D10A ]-induced targeting. The improved gene targeting success rate with short arm vectors is likely facilitated by genotyping using PCR across the short arm.

To address the possibility of tandem or off-target insertions of the vector, we tested clones from 20 genes using quantitative PCR with a neomycin probe. The large majority of clones (73%) have only a single copy of the vector inserted in the genome (Supplemental Fig. 3). The incidence of multi-copy insertion of the vector (27%) is elevated compared to what has been observed with conventional targeting in our hands (202 of 9496; 2.1%). Thus, we would strongly advise the use of quantitative PCR or Southern blotting to distinguish heterozygous or homozygous targeted mutations from multi-copy insertions of the targeting vector.

To expedite the adoption of CRISPR-assisted conditional mutagenesis using IKMC vectors, we have implemented a new search function in the EuMMCR website for direct access to CRISPR-amenable vectors and matched sgRNAs (https://www.eummcr.org/crispr/search). A total of 12,133 genes, representing 81% of the IKMC conditional resource, have conditional vectors that contain a requisite ‘NGG’ PAM sequence within or overlapping the recombineered deletions. Of these, 89% have a target sequence that is unique in the genome, enabling the site-specific targeting of approximately 10,800 genes with Cas9. Importantly, the IKMC vector resource was built in a modular way to facilitate the construction of other allele types. The EUCOMMTOOLS project has recently developed a new versatile toolkit for the creation of multifunctional alleles (i.e., fluorescence, marker, site specific recombinases) from the same IKMC vector library^5^.

To our knowledge, the present study represents the first large scale systematic comparison of targeting efficiencies with and without the aid of a site-specific nuclease. Our results show that many difficult-to-target loci are accessible with CRISPR/Cas9[D10A] technology. Importantly, the targeting frequency is substantially higher, reducing the number of colonies that need to be screened. Furthermore, we suggest that the IKMC conditional vector resource could be exploited to generate targeted alleles directly in mouse zygotes^21^,^26^. As the vector shortening protocol has been already adapted to a 96-well high throughput format, thousands of short-armed vectors can be rapidly produced for future community mouse production needs, such as in the International Mouse Phenotyping Consortium.

The advantages of CRISPR for mutagenesis are not to be ignored; however, in light of our data, we expect that researchers will chose to combine CRISPR technology and the extensive IKMC vector resources to engineer conditional or multifunctional alleles in mice.

## Methods

### Linear-Linear Homologous Recombination

All vectors and genotyping primers were obtained from http://www.eummcr.org and are listed in Supplemental File 2. Intermediate vectors were acquired and linearized with AsiSI and purified. pACYC184 (p15a backbone, F. Stewart laboratory) was linearized with EcoRV & SalI was then amplified by PCR, digested with DpnI and purified. GB05-dir was cultured in 3 ml LB/Streptomycin, 30 °C overnight, inoculated into 110 ml LB/Streptomycin with 3 ml. 1.1 ml was aliquoted into 96 well deep box, cultured for 2.5 hours at 30 °C, 25 μl 10% arabinose was added to each well and incubated for 45 mins at 37 °C. Box was spun and pellets washed in cold water 3 times to make cells electrocompetent.

Next, pellets were resuspended in water (45 μl) with 50–200 ng PCR product & 300–800 ng intermediate vector, transferred to cuvette, and electroporated. Following electroporation, 50 μl 2x Recovery Media were added and transferred to plate containing 500 μl Recovery Media, incubated for 70 mins at 37 °C and then inoculated into 750 μl LB/Chloramphenicol/Zeomycin with 250 μl recovery culture at 30 °C for 48 hours. Resulting vector sizes are approximately 6–8 Kb.

Shortened intermediate vectors are then converted to IKMC final targeting vectors (for sequences, see Supplemental File 3) via a two-way Gateway recombineering reaction with pL1L2_Bact_P, as previously reported^6^, selecting for the chloromanphenicol resistance of the p15a backbone.

### PCR Primers

Hybrid 70 mer oligonucleotides were used for linear-linear gap repair. The appending sequences were ACAACTTATATCGTATGGGGC, 3′ end of G5 oligos. TTACGCCCCGCCCTGCCACTC at 3′ end of G3 oligos, after 50 bp homology to the end of the shortened homology arm.

### Bacterial strains

The *E. coli* strains used were: GB05, derived from DH10B by deletion of fhuA, ybcC and recET22, 49. GB05-dir, derived from GB2005 by the PBAD-ETgA operon, was integrated into the ybcC locus in GB2005 to create GB05-dir. The integration ablates expression of ybcC, which encodes a putative exonuclease similar to that encoded by Redα.

### Media

2x Recovery Media: 2xLB & 0.2% Glucose (10 ml 2xLB & 100 μl 20% glucose), 1x Recovery Media: LB & 0.1% Glucose (50 ml LB & 250 μl 20% glucose).

### Selection of gRNAs

gRNA’s targeting loxP deletion regions were found by directly inspecting the genomic sequence in the deleted region for gRNAs. All gRNAs in the deletions were scored by directly summing the number of off-target hits with 0 to 3 mismatches as recorded in the WTSI WGE database (http://www.sanger.ac.uk/htgt/wge). The resulting gRNAs were arranged into pairs ranked by combined score, and the best pair of gRNAs was chosen for each targeting experiment.

### ES cell electroporation

Plasmids were electroporated using the Amaxa Nucleofector system (Lonza). In brief, 2 μg targeting vector and 3 μg of each sgRNA plasmid were combined, ethanol precipitated, washed twice with 70% ethanol and dried. The pellet was resuspended in sterile PBS (21 μl). 1 μl was used to check the quality on an agarose gel and 4 μg of the Cas9 D10A nickase plasmid was added. Cells were washed with PBS, trypsinized and counted. 5 × 10^6^ cells were transferred into a new tube, centrifuged at 175 g for 3 min at room temperature and the pellet was suspended in of Nucleofector solution (100 μl). The plasmid mix was added and transferred to the AMAXA electroporation cuvette and pulsed using the ‘A-23’ AMAXA-2b program for mouse cells. The cells were transferred by disposable Pasteur pipettes (Lonza) to gelatinized 10 cm dishes with JM8 media.

### Genotyping

Design of LRPCR genotyping oligos: The gene-specific long-range PCR genotyping primers used to identify clones were designed as follows: primers were chosen by examining 2 kb of genomic sequence flanking the 5′ and 3′ targeting vector homology arms. This flanking sequence was tiled into all possible sequences between 24 bp and 30 bp in length, with each successive tile were separated by 1 bp. Each tile was scored to ensure its melting point was below 64 °C, that it had sufficiently high GC-content (number of G’s and C’s together >10), that it minimized triple ‘runs’ of nucleotides such as “AAA” etc., and that it minimized occurrences of self-annealing ends (“GG”, “CC”). Candidate high-scoring tiles (primers) were then aligned to the mouse genome using the Exonerate aligner. Candidate primers which had few, poorly aligning matches in other parts of the genome were scored higher than those with many closely-matching alignments. This yielded three top-scoring candidates gene-specific primers in the 5′ homology arm and 3 candidate primers in the 3′ homology arm. See Supplemental File 2 for all primer sequences.

Cell Lysis: Picked colonies were duplicated and the next day duplicated plates were washed with PBS and frozen at −80 °C. The next day, plates were placed on ice and 30 μl/well of 2× lysis buffer was added to each well (Multidrop, black cartridge) and put directly on ice. 2 μl proteinase K (20 mg/ml) per well was subsequently added and plates were wrapped with foil lids and shaken for one minute on plate shaker, centrifuged for one minute at 400 rpm and incubated overnight at 60 °C. The next day, the plates were shaken for one day on plate shaker and spun shortly. 15 μl of this mixture was then pipetted to a 384 plate, covered with transparent film, and centrifuged for one minute at 1200 rpm. The Proteinase K was then heat-inactivated in a thermocycler: 90 °C for two minutes, spun and then put on ice. Each of a horizontal row 2 μl DNA was from a 96 well plate and was then mixed with 2 μl 2x-cresol-loading buffer and run on a 96-Gel (min 130 V, 30) to check DNA integrity and concentration.

Long-range PCR (LRPCR): For 3′ LRPCR (binds in the vector 3′ sequence ) with two different gene-specific reverse primers (GR3, GR4 ) two separate reaction mixtures are combined each a universal forward primer (J2, GCAATAGCATCACAAATTTCACAAATAAAGCA). The 5′ LRPCR is also combined in two separate reaction mixtures with a universal reverse primer (LAR3, CACAACGGGTTCTTCTGTTAGTCC) with two different gene-specific forward primers (GF3, GF4). Subsequently, up to 9 kb long amplified fragments are sequenced from the vector ends using Sanger sequencing. In addition, the complete LRPCR sequences of ten individual clones from four separate gene targeting experiments was examined for fidelity to the reference sequence (Supplemental File 4). From these primary sequences, which encompass the homologous arms and neighboring genomic DNA, we could not identify any errors that may have arisen during homologous recombination.

Genotyping reactions were performed with LongAmp (NEB). Genotyping was carried out on 384 well plates using 10 μl reaction volumes as follows: Universal primer (100 pmol) 0.09 μl, DNA (50 ng, approximated from gel), 5x Buffer 2 μl, 5 mM dNTP 0.5 μl, 100% DMSO 0.2 μl, Polymerase 0.4 μl, water 2.8 μl. Cycling was as follows: 93 °C three minutes, 8 cycles of [94 °C 15 seconds, touchdown from 68 °C to 60 °C 30′′, 65 °C for 4 minutes 30 seconds], then 27 cycles of [94 °C 15 seconds, 58 °C 30 seconds, 65 °C 4 minutes 30 seconds].

### Plasmids

A codon optimized Cas9 [D10A] nickase^20^ was used together with targeting vectors.

### Quantitative PCR

qPCR was performed using the TaqMan Copy Number Assay (Applied Biosystems). NeoR probe (ID Mr00299300_cn) was used with reference Tfrc (4458367). PCR conditions were 95 °C, 5 min, then 38 cycles of [95 °C, 5 sec, 60 °C, 30 sec] on a ViiA 7 Real-Time PCR machine (Applied Biosystems).

## Additional Information

**How to cite this article**: Schick, J. A. *et al*. CRISPR-Cas9 enables conditional mutagenesis of challenging loci. *Sci. Rep.* **6**, 32326; doi: 10.1038/srep32326 (2016).

## Supplementary Material

Supplementary Information

Supplementary Dataset

Supplementary File 3

## Figures and Tables

**Table 1:** Large scale targeting of previously untargeted IKMC genes with dual-nickase CRISPR.

Vector	Genes	Colonies screened	Correctly targeted colonies (%)	Correctly targeted genes (%)
Long arm	75	2496	590 (25)	35 (47)
Short arm	179	5952	964 (16)	111 (62)

## References

[b1] Infrafrontier Consortium. INFRAFRONTIER—providing mutant mouse resources as research tools for the international scientific community. Nucleic Acids Research 43, D1171–D1175 (2015). 25414328 10.1093/nar/gku1193 PMC4383977

[b2] Brown S. D. & Moore M. W. The International Mouse Phenotyping Consortium: past and future perspectives on mouse phenotyping. Mamm Genome 23, 632–640 (2012). 22940749 10.1007/s00335-012-9427-x PMC3774932

[b3] Smedley D., Salimova E. & Rosenthal N. Cre recombinase resources for conditional mouse mutagenesis. Methods 53, 411–416 (2011). 21195764 10.1016/j.ymeth.2010.12.027

[b4] Bradley A. . The mammalian gene function resource: the International Knockout Mouse Consortium. Mamm Genome 23, 580–586 (2012). 22968824 10.1007/s00335-012-9422-2 PMC3463800

[b5] Rosen B., Schick J. & Wurst W. Beyond knockouts: the International Knockout Mouse Consortium delivers modular and evolving tools for investigating mammalian genes. Mamm Genome (2015). 10.1007/s00335-015-9598-3 26340938

[b6] Skarnes W. C. . A conditional knockout resource for the genome-wide study of mouse gene function. Nature 474, 337–342 (2011). 21677750 10.1038/nature10163 PMC3572410

[b7] Andreasson C. . Direct cloning of isogenic murine DNA in yeast and relevance of isogenicity for targeting in embryonic stem cells. Plos One 8, e74207 (2013). 24058528 10.1371/journal.pone.0074207 PMC3772885

[b8] Szostak J. W., Orr-Weaver T. L., Rothstein R. J. & Stahl F. W. The double-strand-break repair model for recombination. Cell 33, 25–35 (1983). 6380756 10.1016/0092-8674(83)90331-8

[b9] Sung P. & Klein H. Mechanism of homologous recombination: mediators and helicases take on regulatory functions. Nat Rev Mol Cell Biol. 7, 739–750 (2006). 16926856 10.1038/nrm2008

[b10] Cong L. . Multiplex genome engineering using CRISPR/Cas systems. Science 339, 819–823 (2013). 23287718 10.1126/science.1231143 PMC3795411

[b11] Mali P. . RNA-guided human genome engineering via Cas9. Science 339, 823–826 (2013). 23287722 10.1126/science.1232033 PMC3712628

[b12] Hou Z. . Efficient genome engineering in human pluripotent stem cells using Cas9 from Neisseria meningitidis. Proc Natl Acad Sci USA 110, 15644–15649 (2013). 23940360 10.1073/pnas.1313587110 PMC3785731

[b13] Lin S., Staahl B. T., Alla R. K. & Doudna J. A. Enhanced homology-directed human genome engineering by controlled timing of CRISPR/Cas9 delivery. Elife 3, e04766 (2014). 25497837 10.7554/eLife.04766 PMC4383097

[b14] Chen Y. . Engineering Human Stem Cell Lines with Inducible Gene Knockout using CRISPR/Cas9. Cell Stem Cell 17, 233–244 (2015). 26145478 10.1016/j.stem.2015.06.001 PMC4530040

[b15] Zhang Y., Vanoli F., LaRocque J. R., Krawczyk P. M. & Jasin M. Biallelic targeting of expressed genes in mouse embryonic stem cells using the Cas9 system. Methods 69, 171–178 (2014). 24929070 10.1016/j.ymeth.2014.05.003 PMC4405113

[b16] Wang H. . One-step generation of mice carrying mutations in multiple genes by CRISPR/Cas-mediated genome engineering. Cell 153, 910–918 (2013). 23643243 10.1016/j.cell.2013.04.025 PMC3969854

[b17] Shen B. . Efficient genome modification by CRISPR-Cas9 nickase with minimal off-target effects. Nat Methods 11, 399–402 (2014). 24584192 10.1038/nmeth.2857

[b18] Ran F. A. . Double nicking by RNA-guided CRISPR Cas9 for enhanced genome editing specificity. Cell 154, 1380–1389 (2013). 23992846 10.1016/j.cell.2013.08.021 PMC3856256

[b19] Hodgkins A. . WGE: a CRISPR database for genome engineering. Bioinformatics 31, 3078–3080 (2015). 25979474 10.1093/bioinformatics/btv308 PMC4565030

[b20] Ran F. A. . Genome engineering using the CRISPR-Cas9 system. Nat Protoc 8, 2281–2308 (2013). 24157548 10.1038/nprot.2013.143 PMC3969860

[b21] Yang H. . One-step generation of mice carrying reporter and conditional alleles by CRISPR/Cas-mediated genome engineering. Cell 154, 1370–1379 (2013). 23992847 10.1016/j.cell.2013.08.022 PMC3961003

[b22] Beumer K. J., Trautman J. K., Mukherjee K. & Carroll D. Donor DNA Utilization during Gene Targeting with Zinc-finger Nucleases. G3 (Bethesda) (2013). 10.1534/g3.112.005439 PMC3618352 23550125

[b23] Li K., Wang G., Andersen T., Zhou P. & Pu W. T. Optimization of Genome Engineering Approaches with the CRISPR/Cas9 System. Plos One 9, e105779 (2014). 25166277 10.1371/journal.pone.0105779 PMC4148324

[b24] Fu J. . Full-length RecE enhances linear-linear homologous recombination and facilitates direct cloning for bioprospecting. Nat Biotechnol. 30, 440–446 (2012). 22544021 10.1038/nbt.2183

[b25] Deng C. & Capecchi M. R. Reexamination of gene targeting frequency as a function of the extent of homology between the targeting vector and the target locus. Mol Cell Biol. 12, 3365–3371 (1992). 1321331 10.1128/mcb.12.8.3365-3371.1992 PMC364584

[b26] Zhou J. . Dual sgRNAs facilitate CRISPR/Cas9-mediated mouse genome targeting. FEBS J 281, 1717–1725 (2014). 24494965 10.1111/febs.12735

