# Supplementary material for: CRISPR-Cas9 enables conditional mutagenesis of challenging loci
Source: Sci Rep. 2016 Sep 1;6:32326. doi: 10.1038/srep32326 (PMC5007477; doi:10.1038/srep32326)
Supplement: Supplementary Information [file srep32326-s1.pdf]

Supplementary Information

CRISPR-Cas9 enables conditional mutagenesis of challenging loci

Joel A. Schick [1,2§], Claudia Seisenberger [1§], Joachim Beig [1§], Antje Bürger [1], Vivek Iyer [3], Viola Maier [1], Sajith Perera [3] , Barry Rosen [3,4], William C. Skarnes [3*], Wolfgang Wurst [1,5,6,7*]

[§] These authors contributed equally
[*] Authors for correspondence

[1] Institute of Developmental Genetics
Helmholtz Zentrum München
Ingolstädter Landstraße 1
85764 Neuherberg
Germany

[2] Current Address
Institute of Molecular Toxicology and Pharmacology
Helmholtz Zentrum München
Ingolstädter Landstraße 1
85764 Neuherberg
Germany

[3] Wellcome Trust Sanger Institute
Wellcome Trust Genome Campus
Hinxton, Cambridge
UK

[4] AstraZeneca
Darwin Building (Unit 310)
Cambridge Science Park
Milton Road
Cambridge, CB4 0WG
United Kingdom

[5] Technische Universität München-Weihenstephan,
c/o Helmholtz Zentrum München
Ingolstädter Landstr. 1
85764 Neuherberg/Munich
Germany

[6] German Center for Neurodegenerative Diseases (DZNE)
Site Munich
Feodor-Lynen-Strasse 17
81377 Munich
Germany

[7] Munich Cluster for Systems Neurology (SyNergy)
Adolf-Butenandt-Institut
Ludwig-Maximilians-Universität München
Schillerstr. 44
80336 Munich
Germany

Supplemental Table 1

	Genes Electroporated	Successful	Targeting Failures in x Experiments												Failures	Failure Rate	Mean Failures
			1x	2x	3x	4x	5x	6x	7x	8x	9x	10x	11x	12x			
Total	14,886	13,027	888	481	287	126	48	22	4	2				1	1,859	12.49%	1.96

Supplementary Table 1. Failed conventional IKMC targeting experiments and number of repeated attempts. The total number of IKMC gene targetings was tallied and categorized into successful or failed experiments (electroporations). In total, 1,859 gene targeting experiments were classified as failures with most genes only being attempted more than once (971) up to a total of 12 times. A large fraction of genes (888) were attempted only a single time.

Supplementary Figure 1. IKMC intermediate vectors short-arm vector synthesis. A two-step protocol for generation of short-arm conditional targeting vectors is used. IKMC intermediate vectors are recombined with a PCR generated vector backbone via a simple linear-linear recombination strategy (gap repair) followed by a two-way Gateway with pL1L2_Bact_P to generate “knockout first, conditional ready” short arm vectors. G5, G3, small homologies to vector in primers; zeo, zeomycin resistance; PheS, phenylalanine tRNA synthetase; attRx, Gateway compatible recombination site; RecET, Red recombineering system; SA, splice acceptor; LacZ:neo, beta-galactosidase, neomycin phosphotransferase; amp, ampicillin resistance; chl, chloramphenicol resistance; ori, origin of replication.

Supplementary Figure 2. Cas9 dual-nickase strategy improves the mean frequency of correctly targeted ES cell clones. Comparison of the frequency of positive colonies resulting from individual vector electroporations from conventional targeting and Cas9[D10A]-assisted targeting reveals a significant increase in the frequency of positive colonies with the Cas9[D10A] strategy ($p < 0.0001$, two-tailed t test). Red data points are long arm (~5 kb) and blue data points are short arm (~1 kb) vectors.

Supplementary Figure 3. Quantitative PCR of neomycin gene following dual-nickase Cas9[D10A]-assisted targeting in ES cells. Quantitative PCR analysis determining number of genomic copies of the neomycin gene from targeting vectors was determined for 24 colonies from each of 20 genes compared to a control gene (Tfrc). Approximately seventy-five percent of clones show a single insertion.

Supplementary File 1. Genotyping schematic and long-range PCR gel pictures for individual clones from ten Cas9[D10A] targeted genes.

Supplementary File 2. A list of all genes, vectors, sgRNA sequences and genotyping primers used in the study.

Supplementary File 3. Genbank vector files for all targeting vectors used in the study.

Supplementary File 4. Direct sequencing results on long-range PCR fragments from ten clones and multiple sequence alignment with the targeting vector, JM8 cell line and reference sequence.

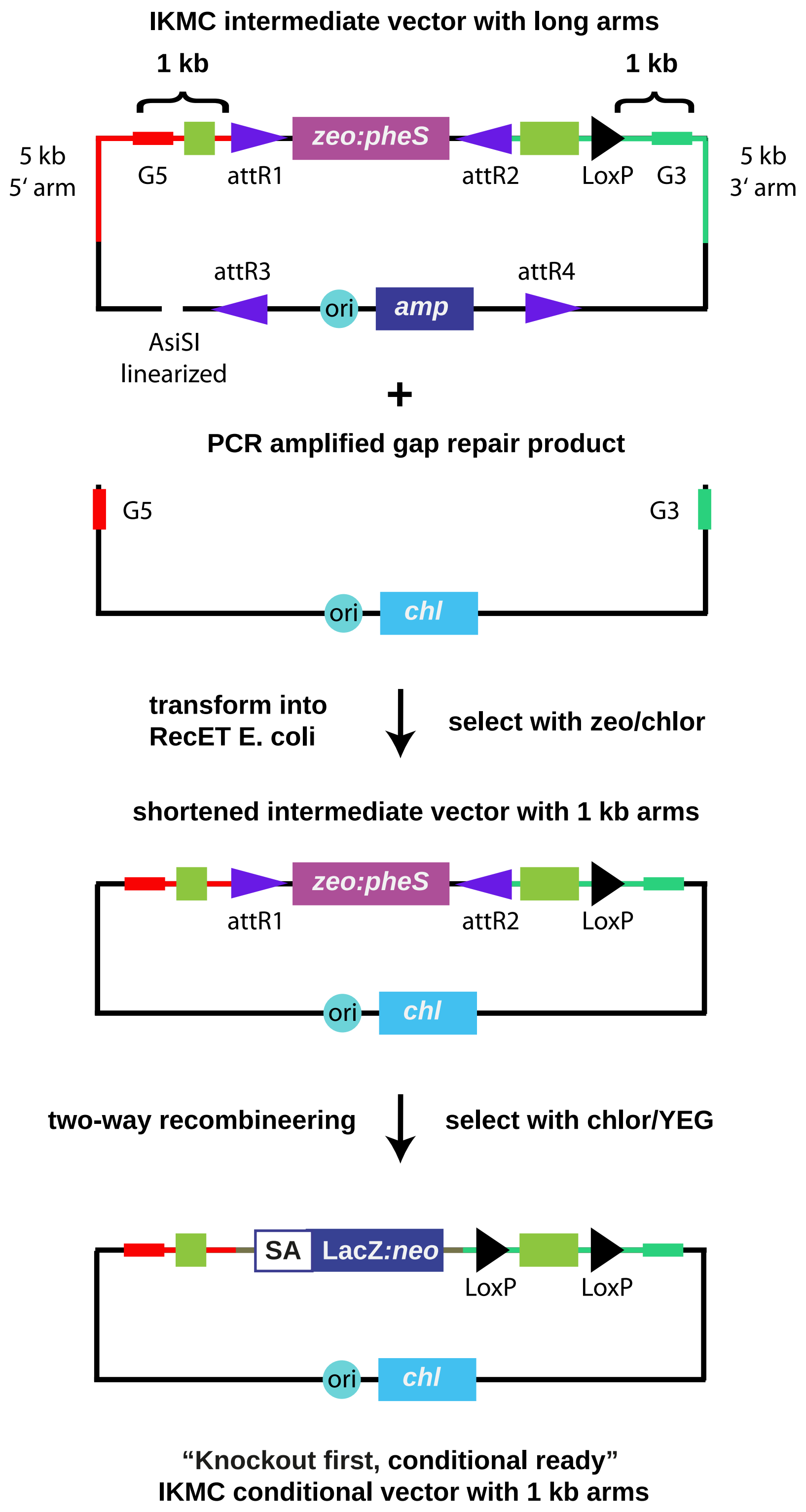
IKMC intermediate vector with long arms
1 kb
1 kb
5 kb
5′ arm
G5
attR1
zeo:pheS
attR2
LoxP
G3
5 kb
3′ arm
attR3
ori
amp
attR4
AsiSI
linearized
+
PCR amplified gap repair product
G5
G3
ori
chl
transform into
RecET E. coli
select with zeo/chlor
shortened intermediate vector with 1 kb arms
zeo:pheS
attR1
attR2
LoxP
ori
chl
two-way recombineering
select with chlor/YEG
SA
LacZ:neo
LoxP
LoxP
ori
chl
“Knockout first, conditional ready”
IKMC conditional vector with 1 kb arms

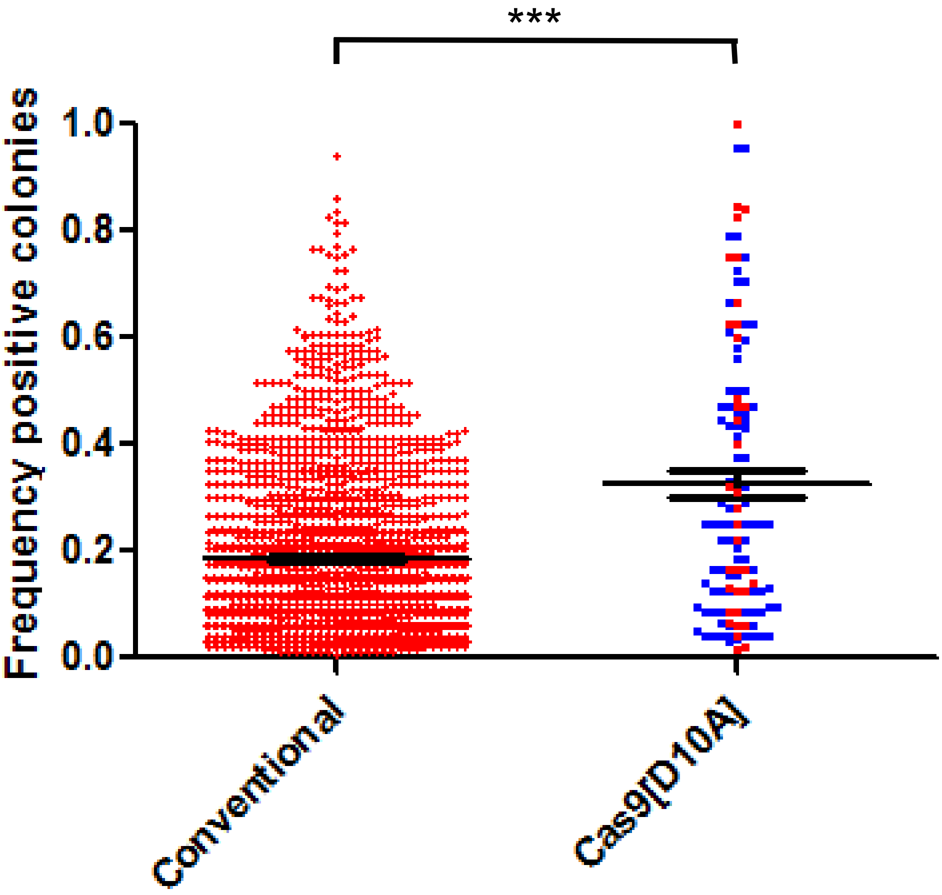

***
Frequency positive colonies
1.0
0.8
0.6
0.4
0.2
0.0
Conventional
Cas9[D10A]

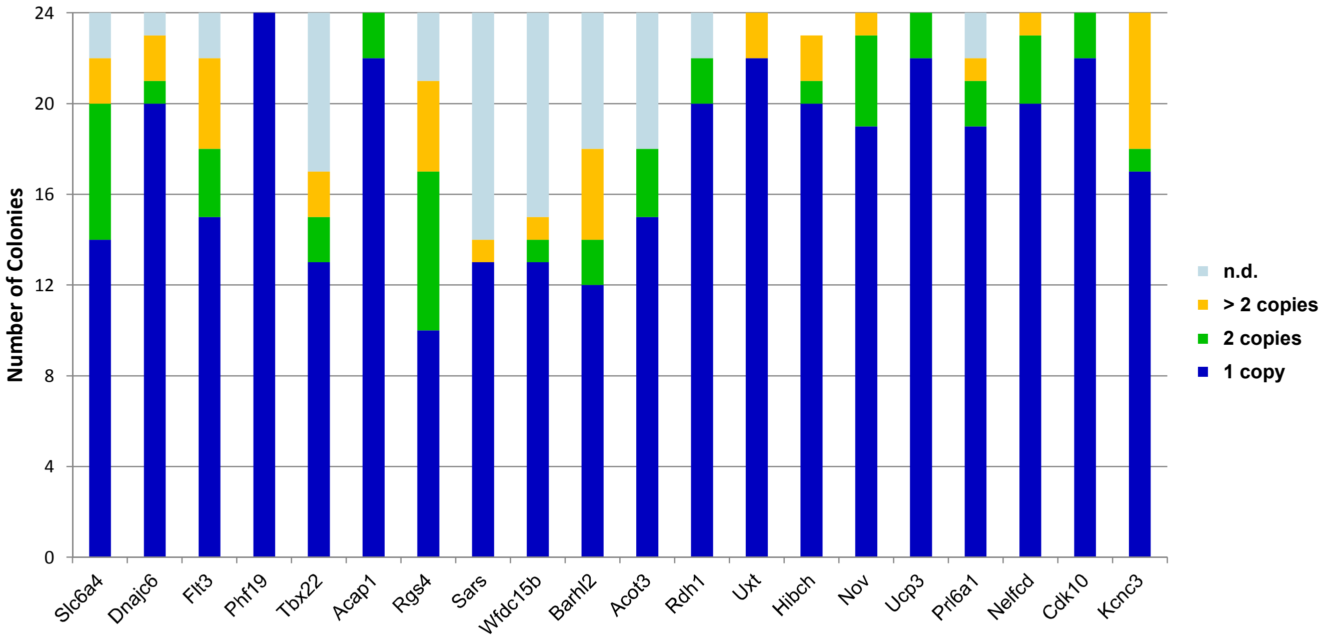

Number of Colonies
24
20
16
12
8
4
0
Slc6a4
Dnajc6
Flt3
Phf19
Tbx22
Acap1
Rgs4
Sars
Wfdc15b
Barhl2
Acot3
Rdh1
Uxt
Hibch
Nov
Ucp3
Prl6a1
Nelfcd
Cdk10
Kcnc3
n.d.
> 2 copies
2 copies
1 copy

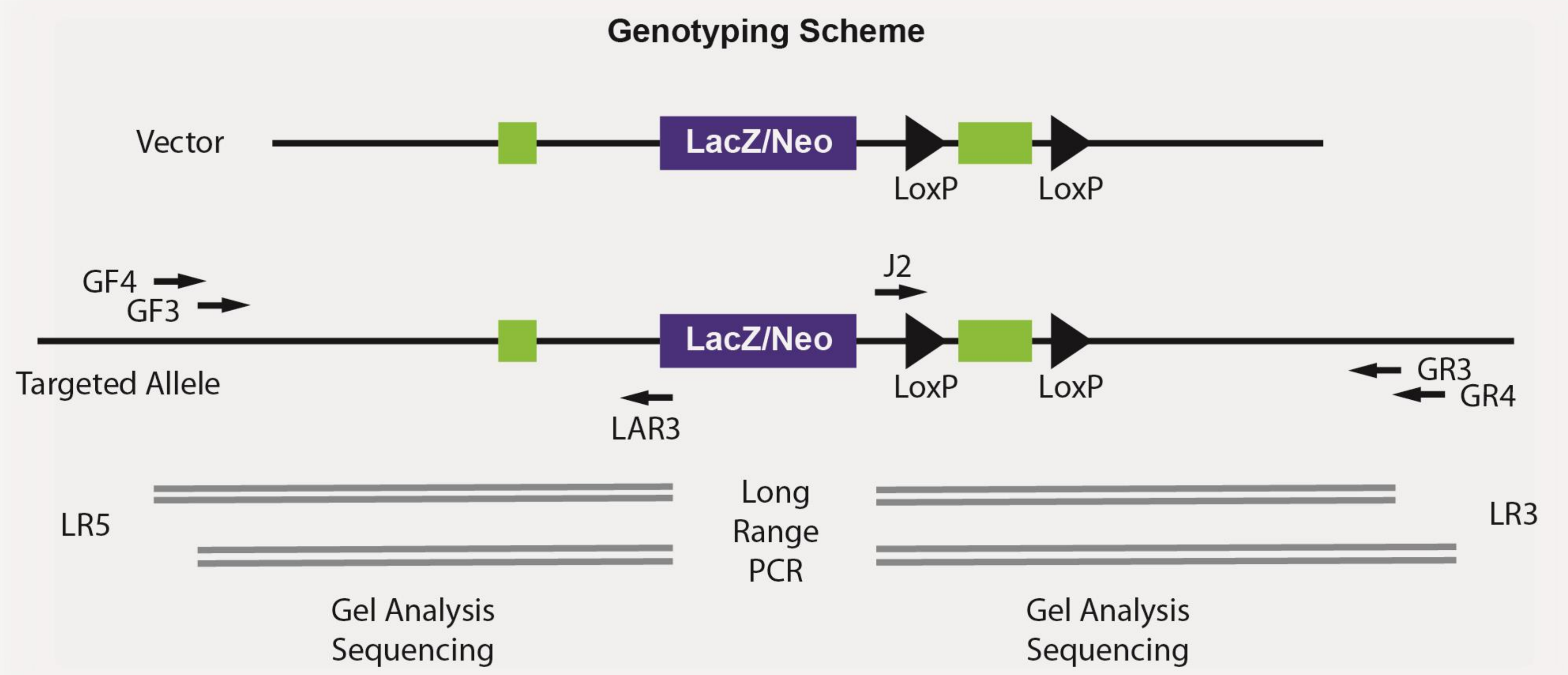

The genotyping scheme used to identify positive colonies following Cas9[D10A]-assisted targeting. In brief, forward primers GF4 and GF3 are unique for each gene and lie in the upstream genomic DNA outside the vector arms and are used together with the universal primer LAR3 (generating LR5). Similarly, downstream primers GR3 and GR4 are outside the vector and are used together with universal primer J2 (generating LR3). For a clone to be identified as 'positive' the' LRPCR must produce bands of the calculated size. LRPCRs with an * were sequenced completely in Supplemental File 4.

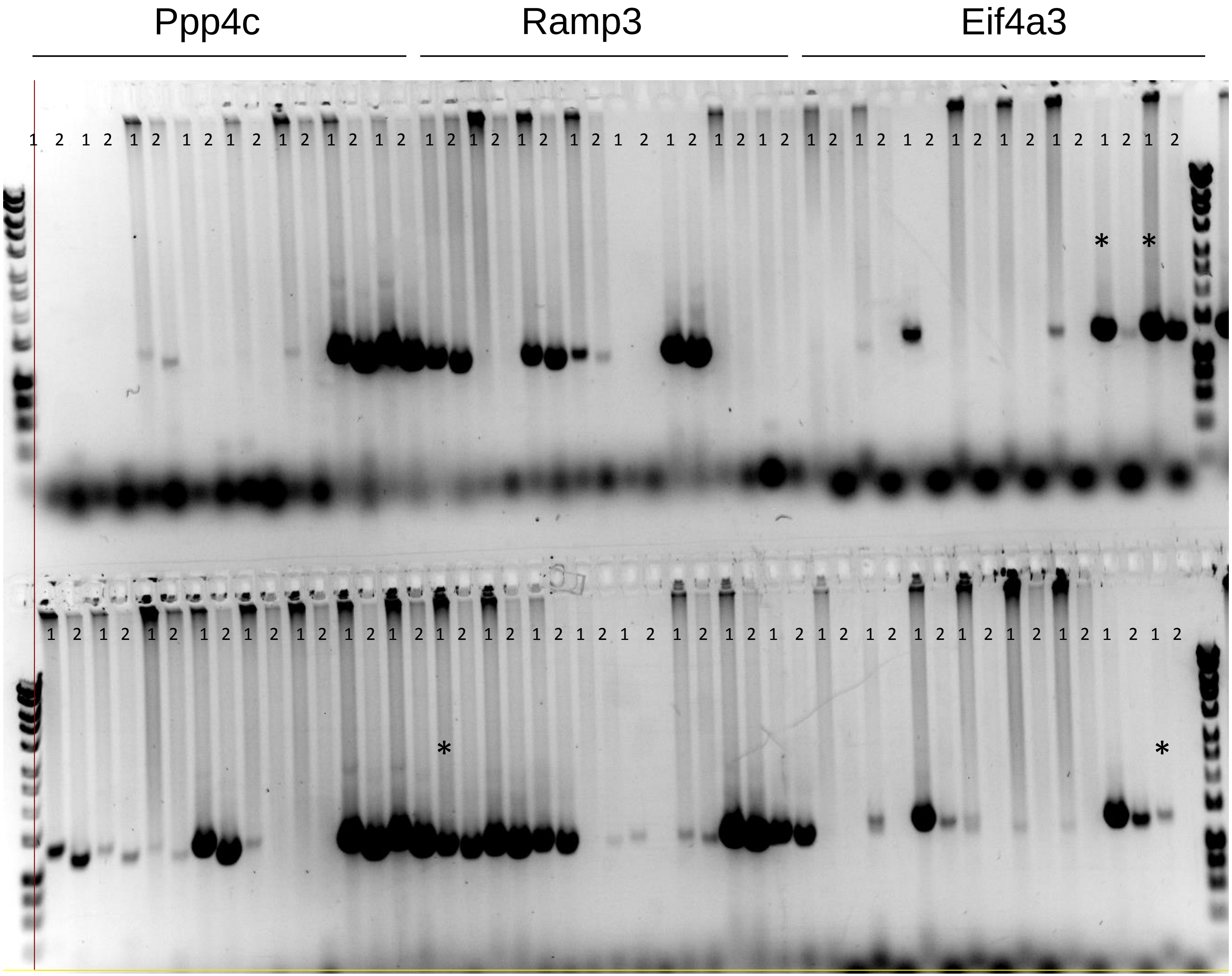

LR5 Gel Lane AB/EF 1: GF3 2: GF4

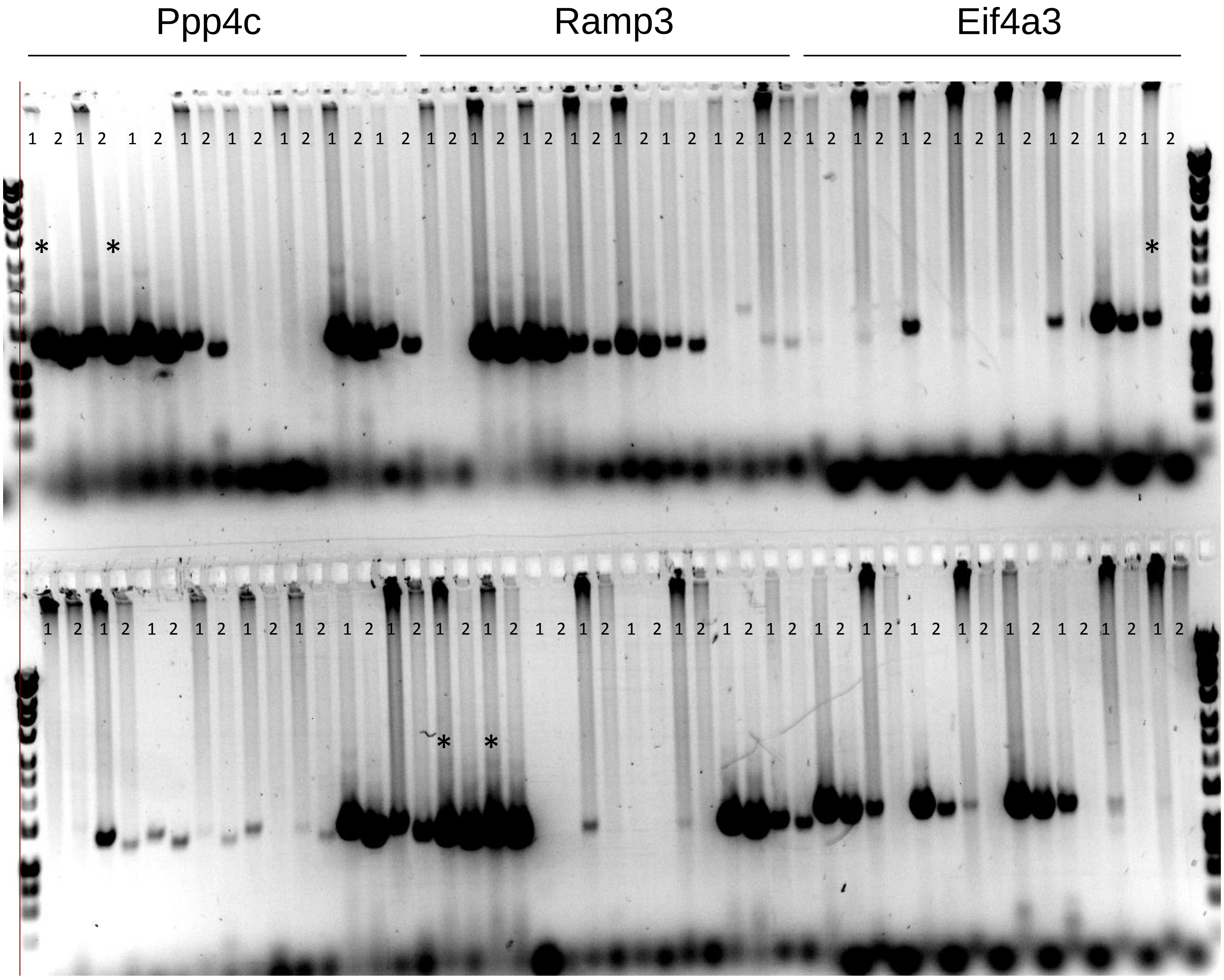

LR5 Gel Lane CD/GH 1: GF3 2: GF4

Ppp4c Ramp3 Eif4a3

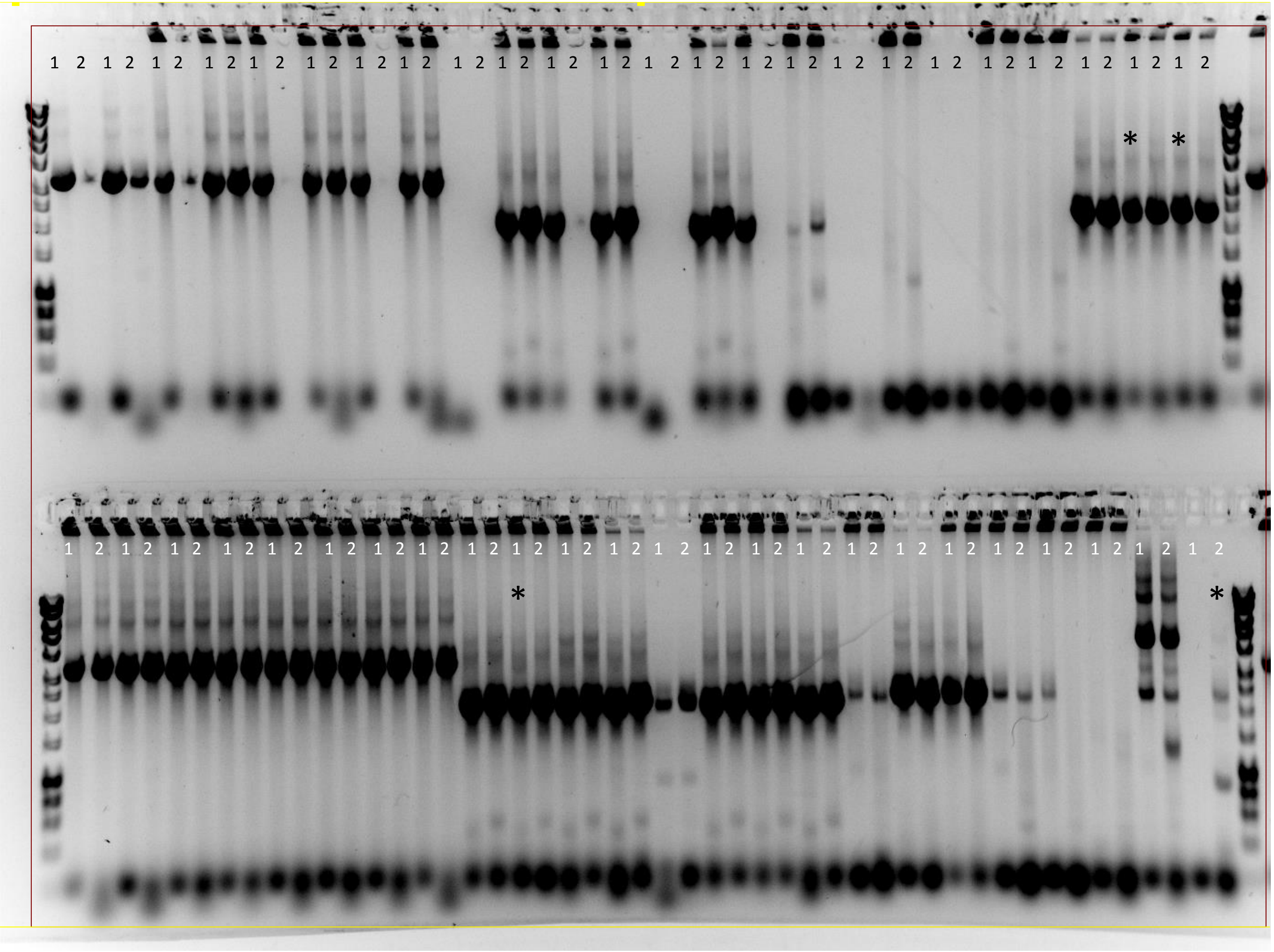

LR3 Gel Lane AB/EF 1: GR3 2: GR4

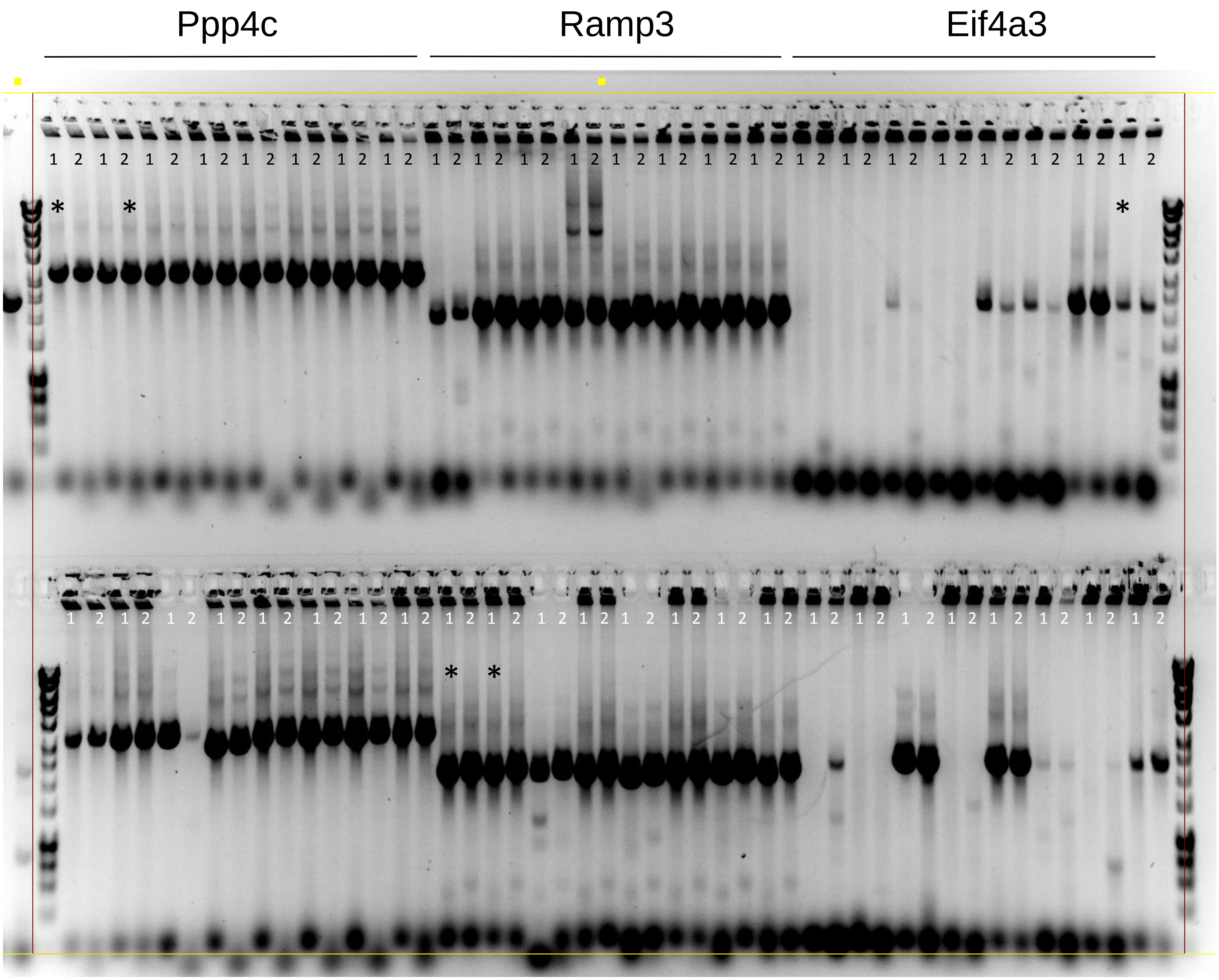

LR3 Gel Lane CD/GH 1: GR3 2: GR4

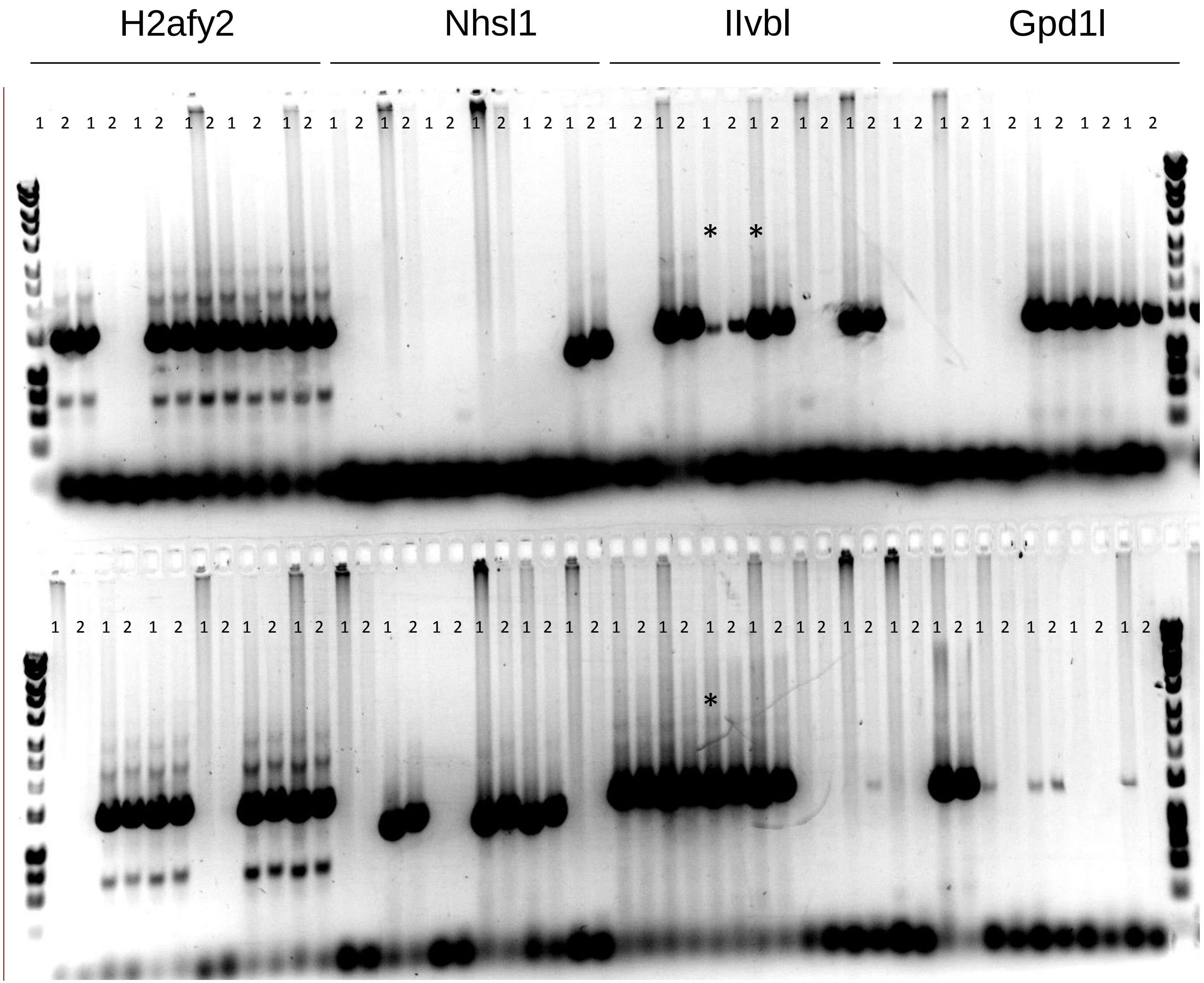

LR5 Gel Lane AB/EF 1: GF3 2: GF4

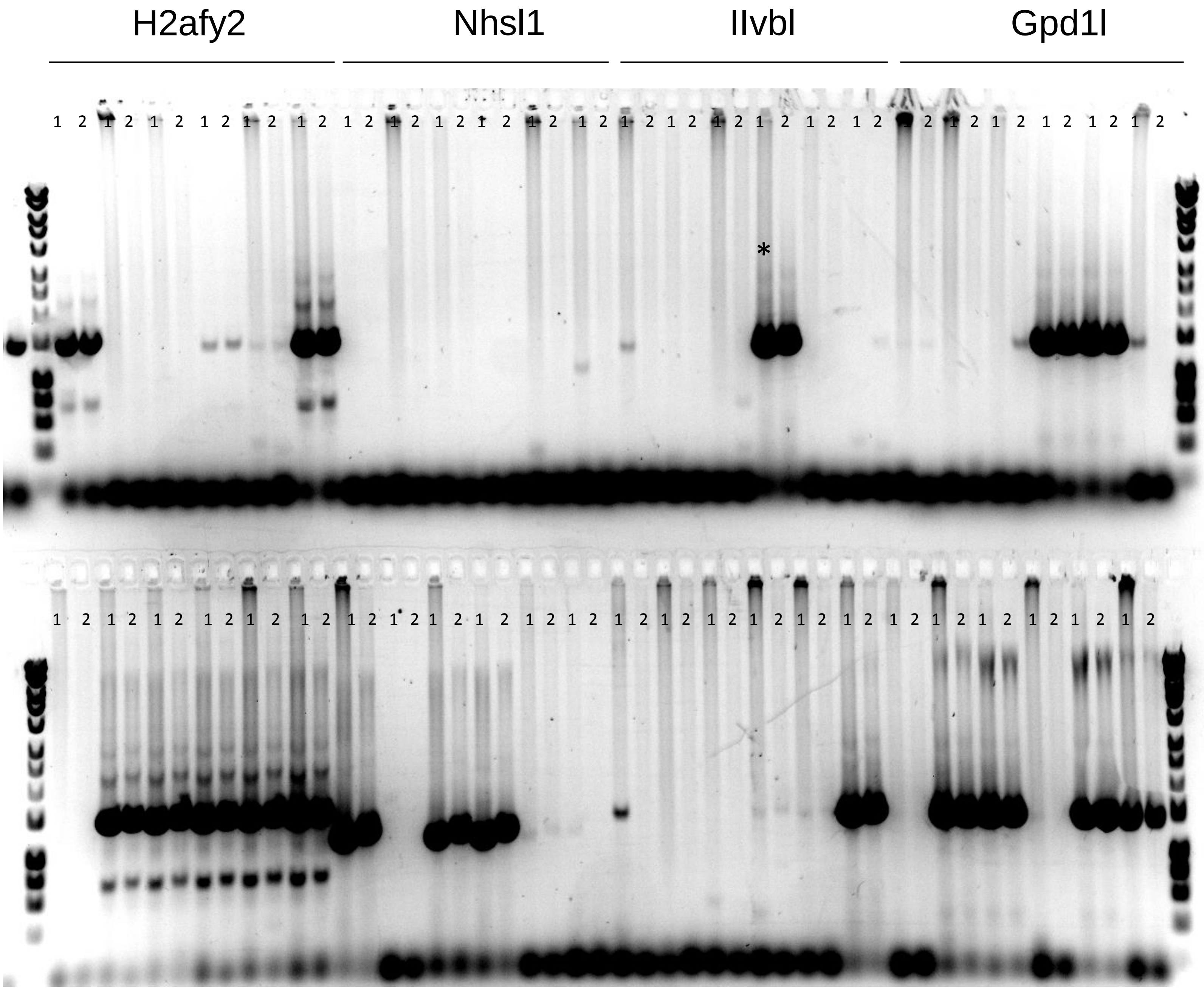

LR5 Gel Lane CD/GH 1: GF3 2: GF4

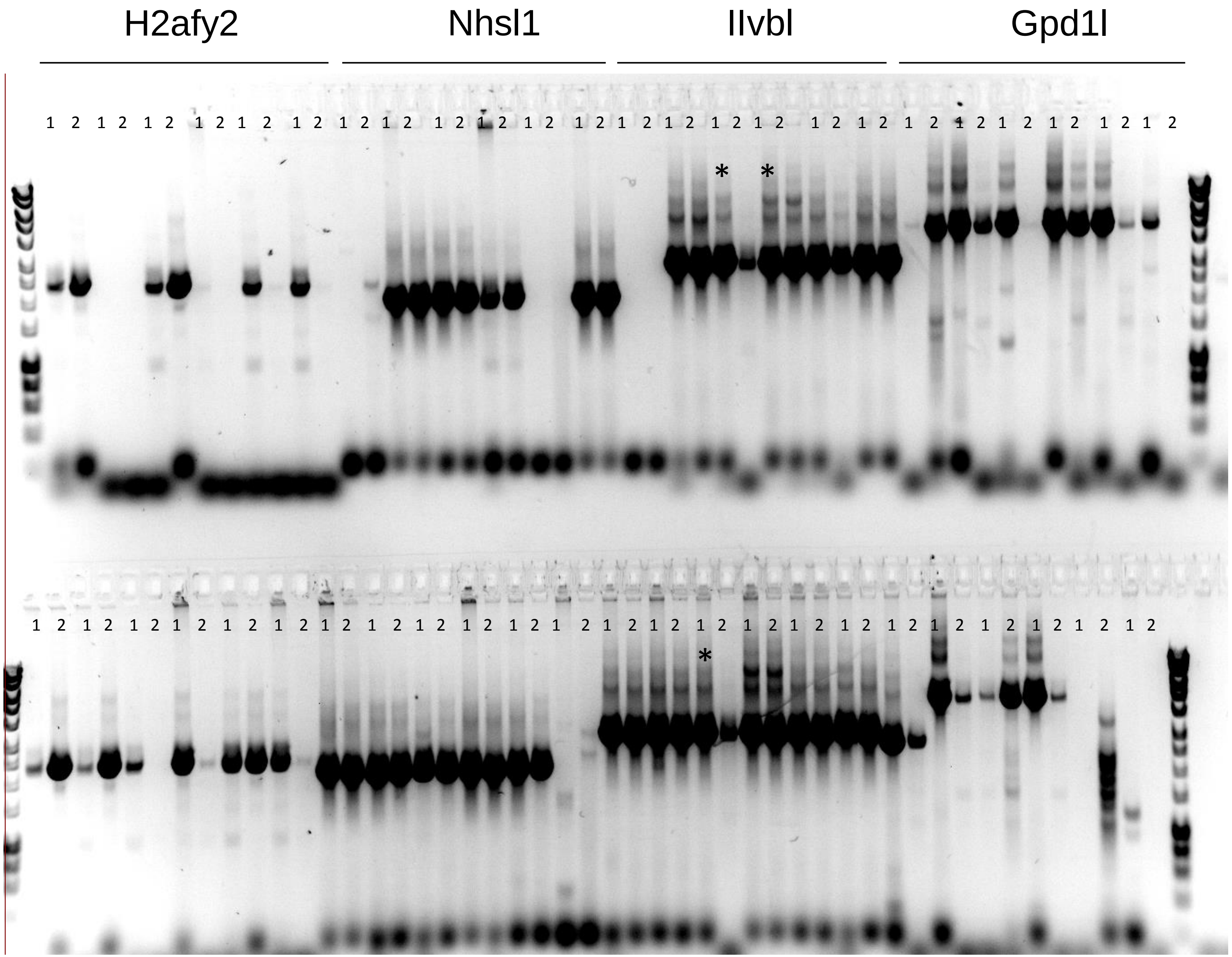

LR3 Gel Lane AB/EF 1: GR3 2: GR4

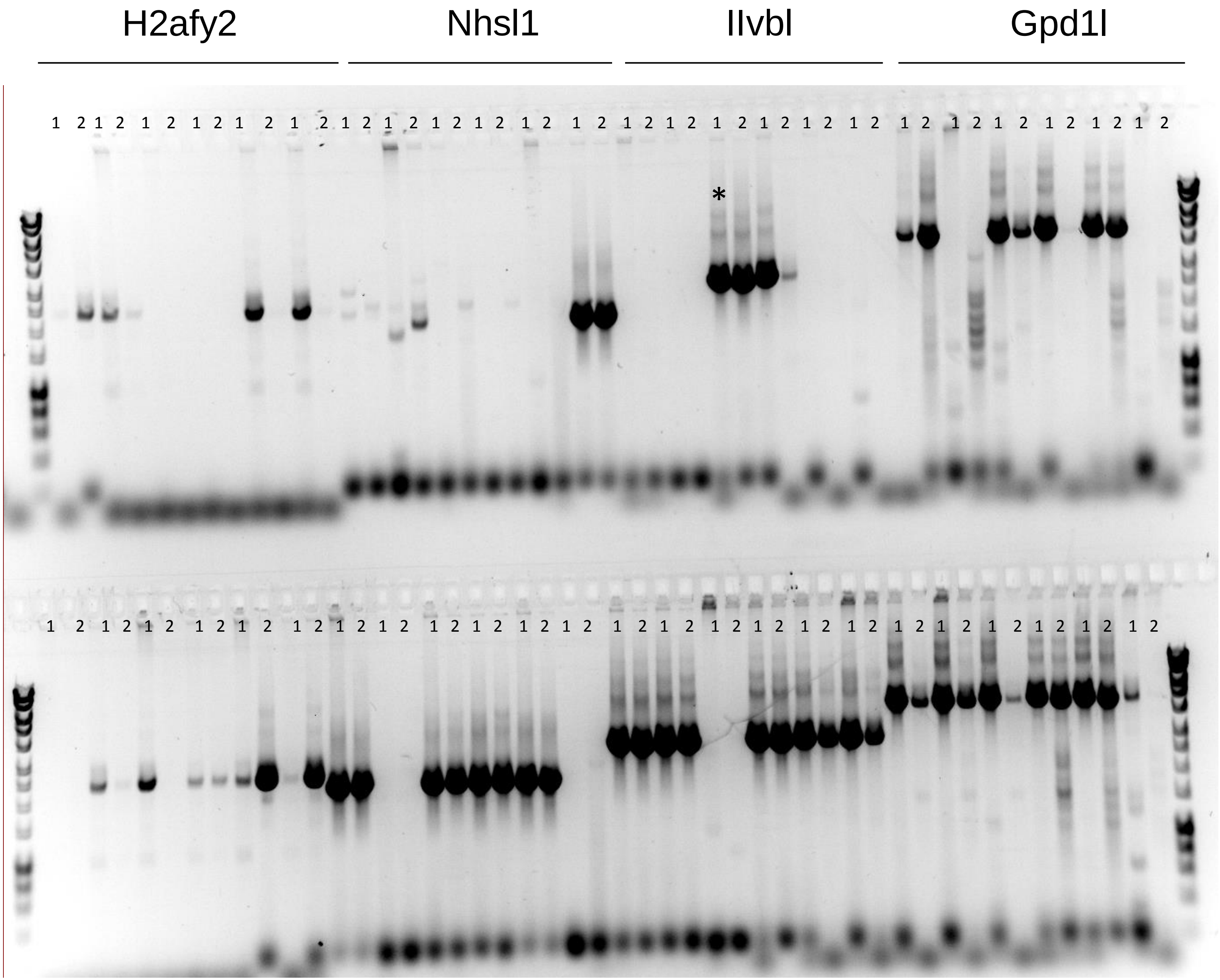

LR3 Gel Lane CD/GH 1: GR3 2: GR4

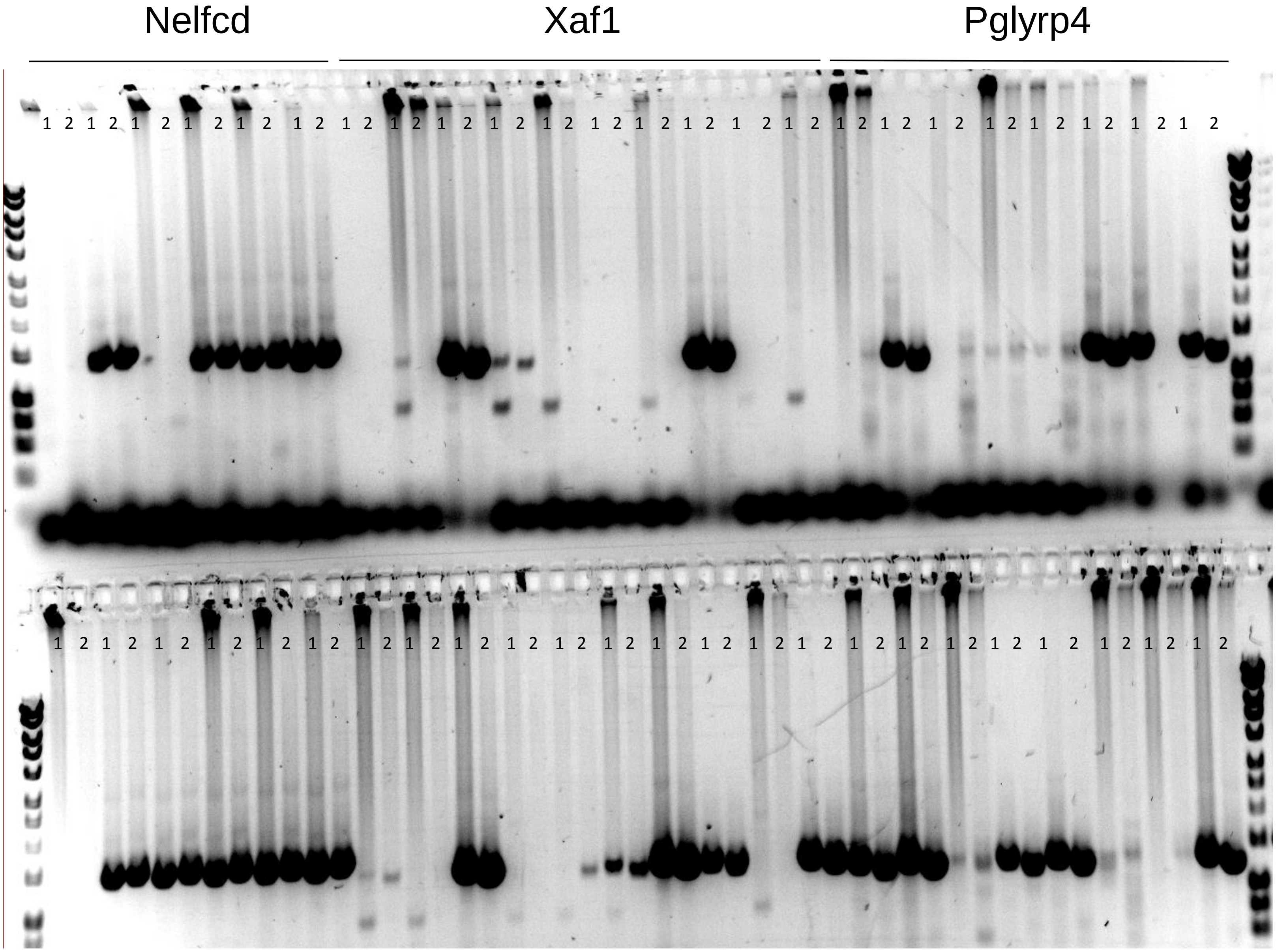

LR5 Gel Lane AB/EF 1: GF3 2: GF4

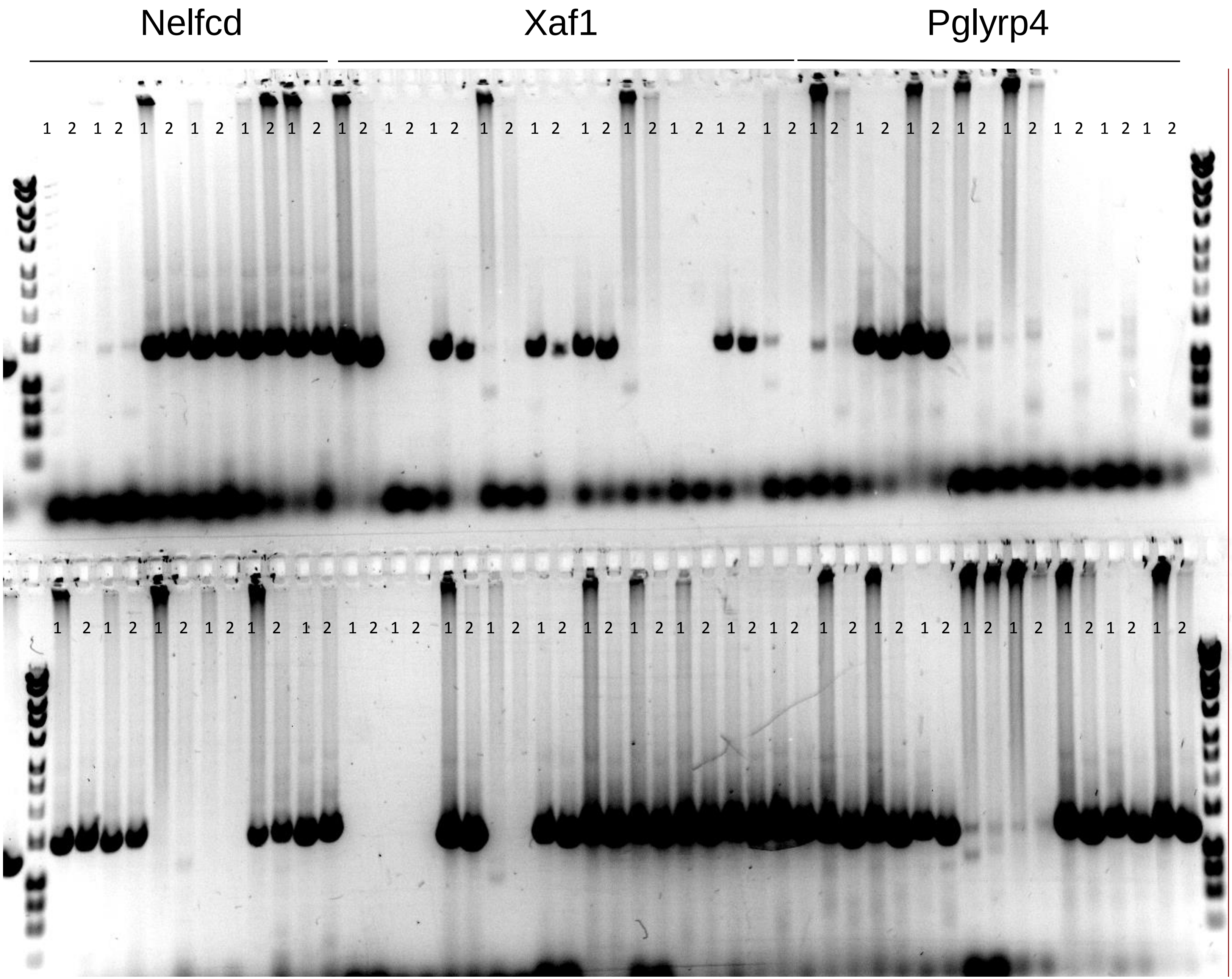

LR5 Gel Lane CD/GH 1: GF3 2: GF4

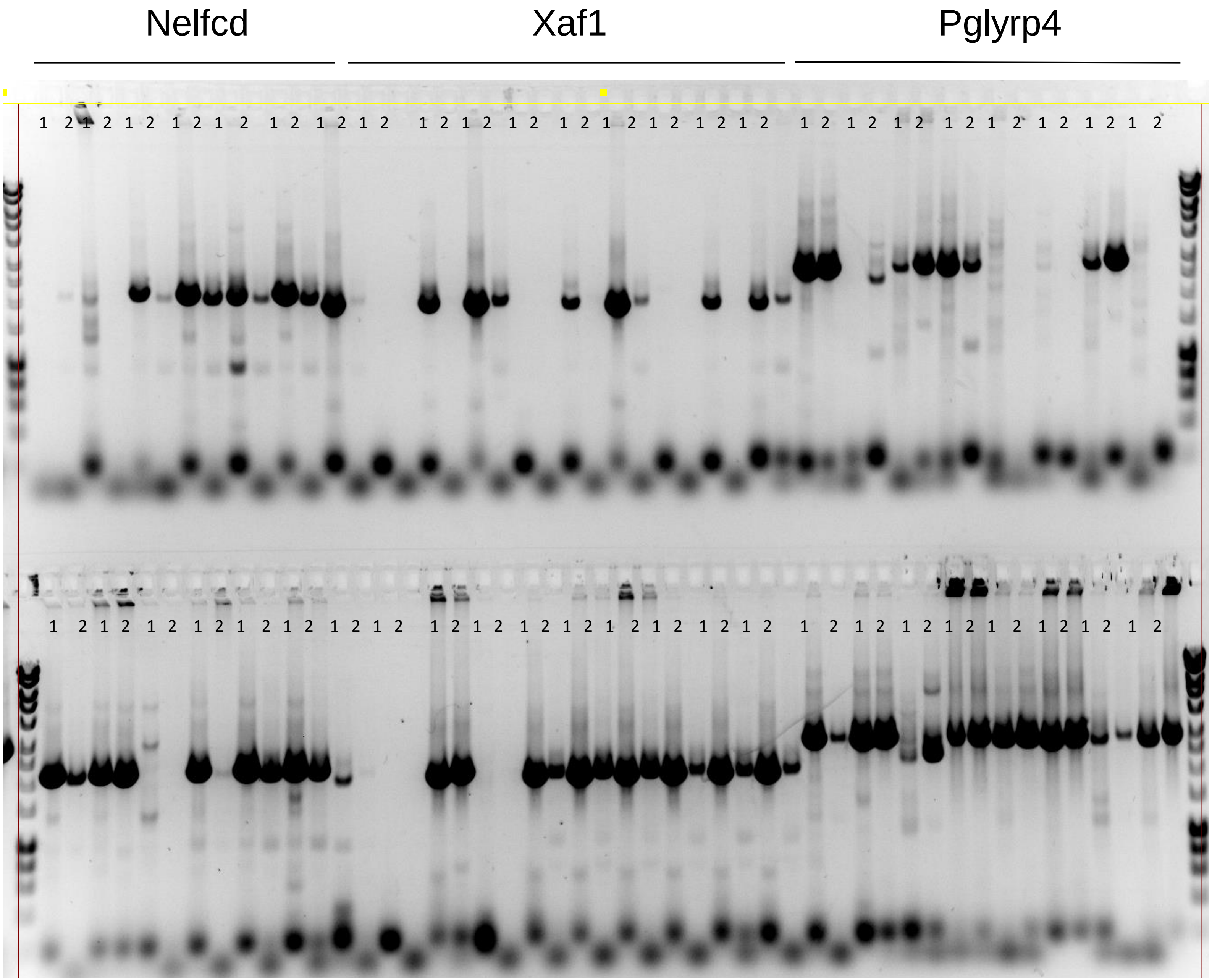

LR3 Gel Lane AB/EF 1: GR3 2: GR4

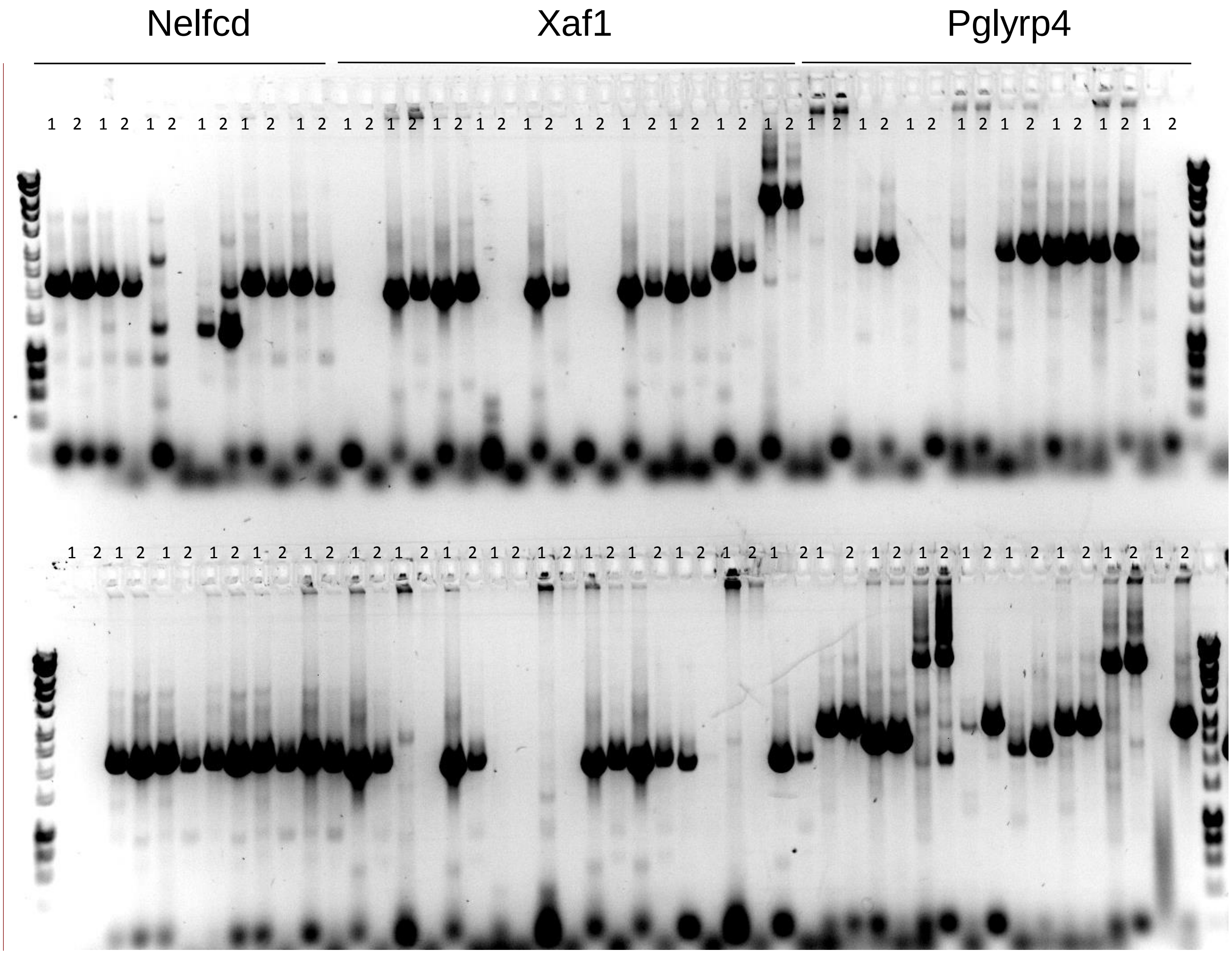

LR3 Gel Lane CD/GH 1: GR3 2: GR4

Ilvbl 5' homology arm

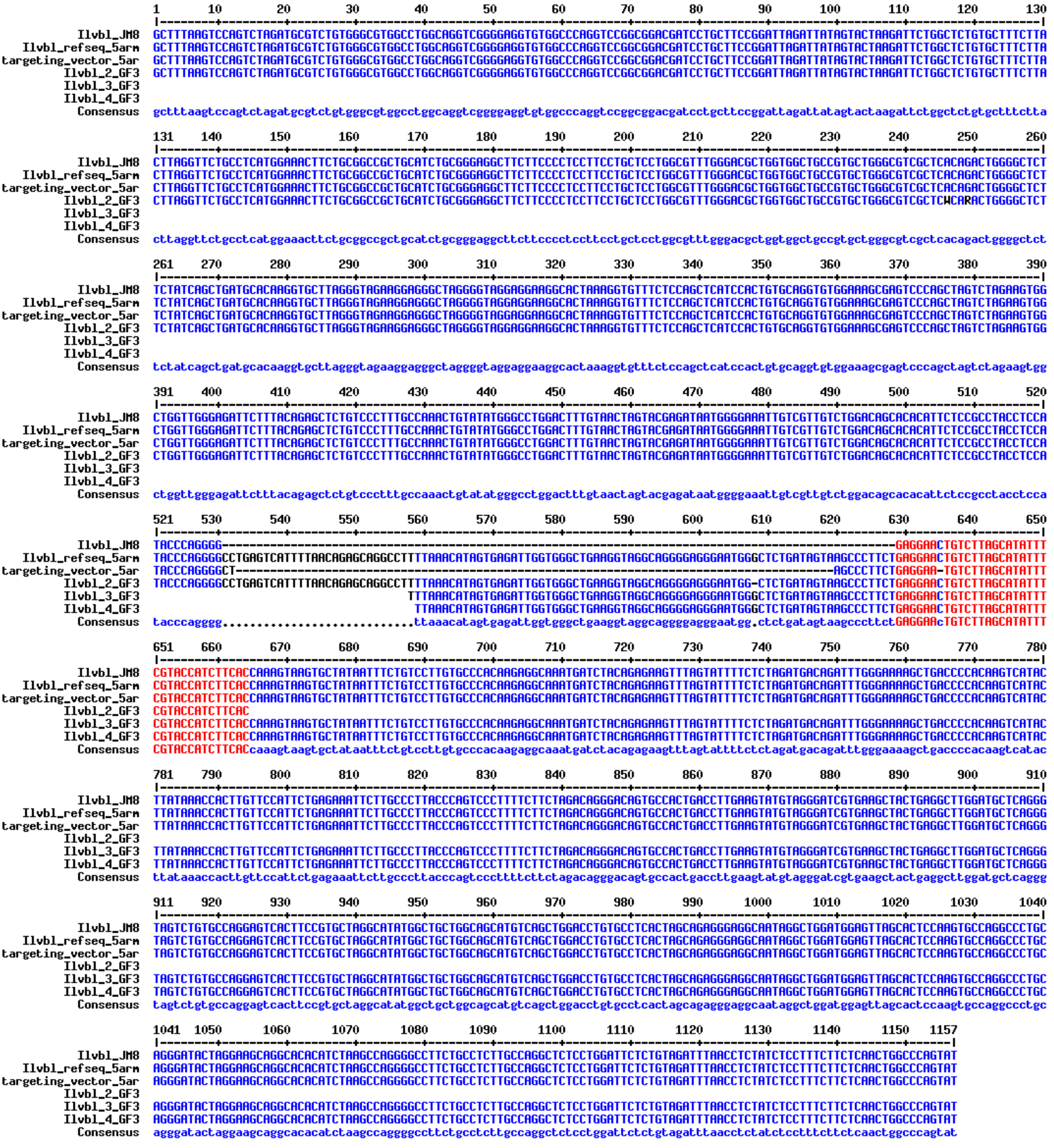

Ilvbl_JM8	direct sequencing JM8 cell line
Ilvbl_refseq_5arm	mouse reference sequence
targeting_vector_5ar	direct sequencing targeting vector arm
Ilvbl_2_GF3	direct sequencing genotyping PCR clone 2
Ilvbl_3_GF3	direct sequencing genotyping PCR clone 3
Ilvbl_4_GF3	direct sequencing genotyping PCR clone 4

Ilvbl 3’ homology arm

```
                     1       10        20        30        40        50        60        70        80        90       100       110       120       130
                     |--------+---------+---------+---------+---------+---------+---------+---------+---------+---------+---------+---------+---------|
           Ilvbl_JM8 TCTTCCTTTTCCTTCTTCTTTTCCTCCATCTCTGCCACCTAGTTATCTCTAGCTTCTCCCTTTCCTTTTTCTTGCCCCTTCCCTCTCAAATATTTAGTGGCCAGATCTATGTGCTAGATAGCAGAATAGG
    Ilvbl_refseq_3arm TCTTCCTTTTCCTTCTTCTTTTCCTCCATCTCTGCCACCTAGTTATCTCTAGCTTCTCCCTTTCCTTTTTCTTGCCCCTTCCCTCTCAAATATTTAGTGGCCAGATCTATGTGCTAGATAGCAGAATAGG
          Ilvbl_2_GR3 TCTTCCTTTTCCTTCTTCTTTTCCTCCATCTCTGCCACCTAGTTATCTCTAGCTTCTCCCTTTCCTTTTTCTTGCCCCTTCCCTCTCAAATATTTAGTGGCCAGATCTATGTGCTAGATAGCAGAATAGG
          Ilvbl_3_GR3 TCTTCCTTTTCCTTCTTCTTTTCCTCCATCTCTGCCACCTAGTTATCTCTAGCTTCTCCCTTTCCTTTTTCTTGCCCCTTCCCTCTCAAATATTTAGTGGCCAGATCTATGTGCTAGATAGCAGAATAGG
          Ilvbl_4_GR3 TCTTCCTTTTCCTTCTTCTTTTCCTCCATCTCTGCCACCTAGTTATCTCTAGCTTCTCCCTTTCCTTTTTCTTGCCCCTTCCCTCTCAAATATTTAGTGGCCAGATCTATGTGCTAGATAGCAGAATAGG
 targeting_vector_3ar
            Consensus tcttccttttccttcttcttttcctccatctctgccacctagttatctctagcttctccctttcctttttcttgccccttccctctcaaatatttagtggccagatctatgtgctagatagcagaatagg

                     131   140       150       160       170       180       190       200       210       220       230       240       250       260
                     |--------+---------+---------+---------+---------+---------+---------+---------+---------+---------+---------+---------+---------|
           Ilvbl_JM8 CAGGAGGTGACATCAGTGGACAAGATATTCAGGTTCTTATGCTCTGGTGTGCAAGAATTGTTTTGAGTGCCAGATCAAGCAAAGGGATTTTGTTTTGTGGCCTTGCCCAAAAAATTCCGGCCCAGATTCT
    Ilvbl_refseq_3arm CAGGAGGTGACATCAGTGGACAAGATATTCAGGTTCTTATGCTCTGGTGTGCAAGAATTGTTTTGAGTGCCAGATCAAGCAAAGGGATTTTGTTTTGTGGCCTTGCCCAAAAAATTCCGGCCCAGATTCT
          Ilvbl_2_GR3 CAGGAGGTGACATCAGTGGACAAGATATTCAGGTTCTTATGCTCTGGTGTGCAAGAATTGTTTTGAGTGCCAGATCAAGCAAAGGGATTTTGTTTTGTGGCCTTGCCCAAAAAATTCCGGCCCAGATTCT
          Ilvbl_3_GR3 CAGGAGGTGACATCAGTGGACAAGATATTCAGGTTCTTATGCTCTGGTGTGCAAGAATTGTTTTGAGTGCCAGATCAAGCAAAGGGATTTTGTTTTGTGGCCTTGCCCAAAAAATTCCGGCCCAGATTCT
          Ilvbl_4_GR3 CAGGAGGTGACATCAGTGGACAAGATATTCAGGTTCTTATGCTCTGGTGTGCAAGAATTGTTTTGAGTGCCAGATCAAGCAAAGGGATTTTGTTTTGTGGCCTTGCCCAAAAAATTCCGGCCCAGATTCT
 targeting_vector_3ar                                                                                            GTTTTGGTGCCTTGCCCAAAAAATTCCGGCCCAGATTCT
            Consensus caggaggtgacatcagtggacaagatattcaggttcttatgctctggtgtgcaagaattgttttgagtgccagatcaagcaaagggattttGTTTTGtgGCCTTGCCCAAAAAATTCCGGCCCAGATTCT

                     261   270       280       290       300       310       320       330       340       350       360       370       380       390
                     |--------+---------+---------+---------+---------+---------+---------+---------+---------+---------+---------+---------+---------|
           Ilvbl_JM8 GTGTAGCCCAGACTG--TTCTGGAACTTGAAAATTCTCTTTCAGCCTCTCAAATGTTGGAATCACAGACAGGTGCCCCAGGTCTAGTCTTGTTAAGACTCCCTGATCTCCTGGTCTTTCTGTCCTCTTCT
    Ilvbl_refseq_3arm GTGTAGCCCAGACTG--TTCTGGAACTTGAAAATTCTCTTTCAGCCTCTCAAATGTTGGAATCACAGACAGGTGCCCCAGGTCTAGTCTTGTTAAGACTCCCTGATCTCCTGGTCTTTCTGTCCTCTTCT
          Ilvbl_2_GR3 GTGTAGCCCAGACTG--TTCTGGAACTTGAAAATTCTCTTTCAGCCTCTCAAATGTTGGAATCACAGACAGGTGCCCCAGGTCTAGTCTTGTTAAGACTCCCTGATCTCCTGGTCTTTCTGTCCTCTTCT
          Ilvbl_3_GR3 GTGTAGCCCAGACTG--TTCTGGAACTTGAAAATTCTCTTTCAGCCTCTCAAATGTTGGAATCACAGACAGGTGCCCCAGGTCTAGTCTTGTTAAGACTCCCTGATCTCCTGGTCTTTCTGTCCTCTTCT
          Ilvbl_4_GR3 GTGTAGCCCAGACTG--TTCTGGAACTTGAAAATTCTCTTTCAGCCTCTCAAATGTTGGAATCACAGACAGGTGCCCCAGGTCTAGTCTTGTTAAGACTCCCTGATCTCCTGGTCTTTCTGTCCTCTTCT
 targeting_vector_3ar GTGTAGCCCAAGACTGATTCTGGAACCTGAAAATCTTCTTTCAGCCTCTCAAATGTTGGAATCACAGACAGGTGCCCCAGGTCTAGTCTTGGTAAGACTCCCTGATCTCCTGGTCTTTCTGTCCTCTTCT
            Consensus GTGTAGCCCAgactg..TTCTGGAACtTGAAAATtcTCTTTCAGCCTCTCAAATGTTGGAATCACAGACAGGTGCCCCAGGTCTAGTCTTGtTAAGACTCCCTGATCTCCTGGTCTTTCTGTCCTCTTCT

                     391   400       410       420       430       440       450       460       470       480       490       500       510       520
                     |--------+---------+---------+---------+---------+---------+---------+---------+---------+---------+---------+---------+---------|
           Ilvbl_JM8 CTCCTTGTCCTCTCTCTCAGTGTCACTTGGAAGACTGGCTCTTTCCCTGACTCTCCTTTACATATTTTTAATTTTAATGGTTTTATGAGAGAGGAGGTAGCTTTGTACCCGTGCTGGCTCAGTATTAGTG
    Ilvbl_refseq_3arm CTCCTTGTCCTCTCTCTCAGTGTCACTTGGAAGACTGGCTCTTTCCCTGACTCTCCTTTACATATTTTTAATTTTAATGGTTTTATGAGAGAGGAGGTAGCTTTGTACCCGTGCTGGCTCAGTATTAGTG
          Ilvbl_2_GR3 CTCCTTGTCCTCTCTCTCAGTGTCACTTGGAAGACTGGCTCTTTCCCTGACTCTCCTTTACATATTTTTAATTTTAATGGTTTTATGAGAGAGGAGGTAGCTTTGTACCCGTGCTGGCTCAGTATTAGTG
          Ilvbl_3_GR3 CTCCTTGTCCTCTCTCTCAGTGTCACTTGGAAGACTGGCTCTTTCCCTGACTCTCCTTTACATATTTTTAATTTTAATGGTTTTATGAGAGAGGAGGTAGCTTTGTACCCGTGCTGGCTCAGTATTAGTG
          Ilvbl_4_GR3 CTCCTTGTCCTCTCTCTCAGTGTCACTTGGAAGACTGGCTCTTTCCCTGACTCTCCTTTACATATTTTTAATTTTAATGGTTTTATGAGAGAGGAGGTAGCTTTGTACCCGTGCTGGCTCAGTATTAGTG
 targeting_vector_3ar CTCCTTGTCCTCTCTCTCAGTGTCACTTGGAAGACTGGCTCTTTCCCTGACTCTCCTTTACATATTTTTAATTTTAATGGTTTTATGAGAGAGGAGGTAGCTTTGTACCCGTGCTGGCTCAGTATTAGTG
            Consensus CTCCTTGTCCTCTCTCTCAGTGTCACTTGGAAGACTGGCTCTTTCCCTGACTCTCCTTTACATATTTTTAATTTTAATGGTTTTATGAGAGAGGAGGTAGCTTTGTACCCGTGCTGGCTCAGTATTAGTG

                     521   530       540       550       560       570       580       590       600       610       620       630       640       650
                     |--------+---------+---------+---------+---------+---------+---------+---------+---------+---------+---------+---------+---------|
           Ilvbl_JM8 TGAGTCCTCTTGTCTCAGTATCCCTGGTGCCGGATTCTCTCTCCTTTTTTAAACCACAGTTCCCCCACCCCCTCCTTTTTTTTCTTGGCCTCATTAACTCCCTATAGTCATGCCTTTAACAGACAAAACAG
    Ilvbl_refseq_3arm TGAGTCCTCTTGTCTCAGTATCCCTGGTGCCGGATTCTCTCTCCTTTTT-AAACCACAGTTCCCCCACCCCCTCCTTTTTTTTCTTGGCCTCATTAACTCCCTATAGTCATGCCTTTAACAGACAAAACAG
          Ilvbl_2_GR3 TGAGTCCTCTTGTCTCAGTATCCCTGGTGCCGGATTCTCTCTCCTTTTT-AAACCACAGTTCCCCCACCCCCTCCTTTTTTTTCTTGGCCTCATTAACTCCCTATAGTCATGCCTTTAACAGACAAAACAG
          Ilvbl_3_GR3 TGAGTCCTCTTGTCTCAGTATCCCTGGTGCCGGATTCTCTCTCCTTTTT-AAACCACAGTTCCCCCACCCCCTCCTTTTTTTTCTTGGCCTCATTAACTCCCTATAGTCATGCCTTTAACAGACAAAACAG
          Ilvbl_4_GR3 TGAGTCCTCTTGTCTCAGTATCCCTGGTGCCGGATTCTCTCTCCTTTTT-AAACCACAGTTCCCCCACCCCCTCCTTTTTTTTCTTGGCCTCATTAACTCCCTATAGTCATGCCTTTAACAGACAAAACAG
 targeting_vector_3ar TGAGTCCTCTTGTCTCAGTATCCCTGGTGCCGGATTCTCTCTCCTTTTTTAAACCACAGTTCCCCCACCCCCTCCTTTTTTTTCTTGGCCTCATTAACTCCCTATAGTCATGCCTTTAACAGACAAAACAG
            Consensus TGAGTCCTCTTGTCTCAGTATCCCTGGTGCCGGATTCTCTCTCCTTTTT.AAACCACAGTTCCCCCACCCCCTCCTTTTTTTTCTTGGCCTCATTAACTCCCTATAGTCATGCCTTTAACAGACAAAACAG

                     651   660       670       680       690       700       710       720       730       740       750       760       770       780
                     |--------+---------+---------+---------+---------+---------+---------+---------+---------+---------+---------+---------+---------|
           Ilvbl_JM8 GTGTTGGCTCTGAGCTCTGACTGCTTCCTGTGTGTCTGTCTTTAGTGAGATTGAGTGTTCCTTGAGGGCAAGGCCTGGGCAGGATTTCTTTTTGTGTCACAGGGTCTAAGAAACTCAAGGCCTGAGGCCT
    Ilvbl_refseq_3arm GTGTTGGCTCTGAGCTCTGACTGCTTCCTGTGTGTCTGTCTTTAGTGAGATTGAGTGTTCCTTGAGGGCAAGGCCTGGGCAGGATTTCTTTTTGTGTCACAGGGTCTAAGAAACTCAAGGCCTGAGGCCT
          Ilvbl_2_GR3 GTGTTGGCTCTGAGCTCTGACTGCTTCCTGTGTGTCTGTCTTTAGTGAGATTGAGTGTTCCTTGAGGGCAAGGCCTGGGCAGGATTTCTTTTTGTGTCACAGGGTCTAAGAAACTCAAGGCCTGAGGCCT
          Ilvbl_3_GR3 GTGTTGGCTCTGAGCTCTGACTGCTTCCTGTGTGTCTGTCTTTAGTGAGATTGAGTGTTCCTTGAGGGCAAGGCCTGGGCAGGATTTCTTTTTGTGTCACAGGGTCTAAGAAACTCAAGGCCTGAGGCCT
          Ilvbl_4_GR3 GTGTTGGCTCTGAGCTCTGACTGCTTCCTGTGTGTCTGTCTTTAGTGAGATTGAGTGTTCCTTGAGGGCAAGGCCTGGGCAGGATTTCTTTTTGTGTCACAGGGTCTAAGAAACTCAAGGCCTGAGGCCT
 targeting_vector_3ar GTGTTGGCTCTGAGCTCTGACTGCTTCCTGTGTGTCTGTCTTTAGTGAGATTGAGTGTTCCTTGAGGGCAAGGCCTGGGCAGGATTTCTTTTTGTGTCACAGGGTCTAAGAAACTCAAGGCCTGAGGCCT
            Consensus GTGTTGGCTCTGAGCTCTGACTGCTTCCTGTGTGTCTGTCTTTAGTGAGATTGAGTGTTCCTTGAGGGCAAGGCCTGGGCAGGATTTCTTTTTGTGTCACAGGGTCTAAGAAACTCAAGGCCTGAGGCCT

                     781   790       800       810       820       830       840       850       860       870       880       890       900       910
                     |--------+---------+---------+---------+---------+---------+---------+---------+---------+---------+---------+---------+---------|
           Ilvbl_JM8 AGTGTGTAGGGAGACAGGAGGCACTGCAAGATCCGAATGGGTAACATTAACAACCACAGCCTGCATTTTGTAAAGTGTTTCTCTCCTTCCCTTGCAATGGTGGTGGAGATGGAACCTGAGGCTTCATATG
    Ilvbl_refseq_3arm AGTGTGTAGGGAGACAGGAGGCACTGCAAGATCCGAATGGGTAACATTAACAACCACAGCCTGCATTTTGTAAAGTGTTTCTCTCCTTCCCTTGCAATGGTGGTGGAGATGGAACCTGAGGCTTCATATG
          Ilvbl_2_GR3 AGTGTGTAGGGAGACAGGAGGCACTGCAAGATCCGAATGGGTAACATTAACAACCACAGCCTGCATTTTGTAAAGTGTTTCTCTCCTTCCCTTGCAATGGTGGTGGAGATGGAACCTGAGGCTTCATATG
          Ilvbl_3_GR3 AGTGTGTAGGGAGACAGGAGGCACTGCAAGATCCGAATGGGTAACATTAACAACCACAGCCTGCATTTTGTAAAGTGTTTCTCTCCTTCCCTTGCAATGGTGGTGGAGATGGAACCTGAGGCTTCATATG
          Ilvbl_4_GR3 AGTGTGTAGGGAGACAGGAGGCACTGCAAGATCCGAATGGGTAACATTAACAACCACAGCCTGCATTTTGTAAAGTGTTTCTCTCCTCCCCTTGCAATGGTGGTGGAGATGGAACCTGAGGCTTCATATG
 targeting_vector_3ar AGTGTGTAGGGAGACAGGAGGCACTGCAAGATCCGAATGGGTAACATTAACAACCACAGCCTGCATTTTGTAAAGTGTTTCTCTCCTTCCCTTGCAATGGTGGTGGAGATGGAACCTGAGGCTTCATATG
            Consensus AGTGTGTAGGGAGACAGGAGGCACTGCAAGATCCGAATGGGTAACATTAACAACCACAGCCTGCATTTTGTAAAGTGTTTCTCTCCTtCCCTTGCAATGGTGGTGGAGATGGAACCTGAGGCTTCATATG

                     911   920       930       940       950       960       970       980       990      1000      1010      1020      1030      1040
                     |--------+---------+---------+---------+---------+---------+---------+---------+---------+---------+---------+---------+---------|
           Ilvbl_JM8 TACTAGACCGGGCCTTTGCCGCTGAGCCACACTCCCAGACCACAATGTGCCTTATAACCTAAGACGGTAGATATGGGAACATCCCCATTTTACAGAGGAGGAAGATCAGAATGCTGAAGGAACTGGACCA
    Ilvbl_refseq_3arm TACTAGACCGGGCCTTTGCCGCTGAGCCACACTCCCAGACCACAATGTGCCTTATAACCTAAGACGGTAGATATGGGAACATCCCCATTTTACAGAGGAGGAAGATCAGAATGCTGAAGGAACTGGACCA
          Ilvbl_2_GR3 TACTAGACCGGGCCTTTGCCGCTGAGCCACACTCCCAGACCACAATGTGCCTTATAACCTAAGACGGTAGATATGGGAACATCCCCATTTTACAGAGGAGGAAGATCAGAATGCTGAAGGAACTGGACCA
          Ilvbl_3_GR3 TACTAGACCGGGCCTTTGCCGCTGAGCCACACTCCCAGACCACAATGTGCCTTATAACCTAAGACGGTAGATATGGGAACATCCCCATTTTACAGAGGAGGAAGATCAGAATGCTGAAGGAACTGGACCA
          Ilvbl_4_GR3 TACTAGACCGGGCCTTTGCCGCTGAGCCACACTCCCAGACCACAATGTGCCTTATAACCTAAGACGGTAGATATGGGAACATCCCCATTTTACAGAGGAGGAAGATCAGAATGCTGAAGGAACTGGACCA
 targeting_vector_3ar TACTAGACCGGGCCTTTGCCGCTGAGCCACACTCCCAGACCACAATGTGCCTTATAACCTAAGACGGTAGATATGGGAACATCCCCATTTTACAGAGGAGGAAGATCAGAATGCTGAAGGAACTGGACCA
            Consensus TACTAGACCGGGCCTTTGCCGCTGAGCCACACTCCCAGACCACAATGTGCCTTATAACCTAAGACGGTAGATATGGGAACATCCCCATTTTACAGAGGAGGAAGATCAGAATGCTGAAGGAACTGGACCA

                     1041 1050      1060      1070      1080      1090      1100      1110      1120      1130      1140      1150      1160      1170
                     |--------+---------+---------+---------+---------+---------+---------+---------+---------+---------+---------+---------+---------|
           Ilvbl_JM8 AAGTCACACAGCTGAGGTGCTGTAGGGCTAGGGTCTAACTCACAACCCTAGTTTCTGTGCTGGCATTCTCCACCTGTTAATGTGTTTCGGGGAACTGTGTCAAGTTAGGGTGGCTTCCTGAAGGGTTGGA
    Ilvbl_refseq_3arm AAGTCACACAGCTGAGGTGCTGTAGGGCTAGGGTCTAACTCACAACCCTAGTTTCTGTGCTGGCATTCTCCACCTGTTAATGTGTTTCGGGGAACTGTGTCAAGTTAGGGTGGCTTCCTGAAGGGTTGGA
          Ilvbl_2_GR3 AAGTCACACAGCTGAGGTGCTGTAGGGCTAGGGTCTAACTCACAACCCTAGTTTCTGTGCTGGCATTCTCCACCTGTTAATGTGTTTCGGGGAACTGTGTCAAGTTAGGGTGGCTTCCTGAAGGGTTGGA
          Ilvbl_3_GR3 AAGTCACACAGCTGAGGTGCTGTAGGGCTAGGGTCTAACTCACAACCCTAGTTTCTGTGCTGGCATTCTCCACCTGTTAATGTGTTTCGGGGAACTGTGTCAAGTTAGGGTGGCTTCCTGAAGGGTTGGA
          Ilvbl_4_GR3 AAGTCACACAGCTGAGGTGCTGTAGGGCTAGGGTCTAACTCACAACCCTAGTTTCTGTGCTGGCATTCTCCACCTGTTAATGTGTTTCGGGGAACTGTGTCAAGTTAGGGTGGCTTCCTGAAGGGTTGGA
 targeting_vector_3ar AAGTCACACAGCTGAGGTGCTGTAGGGCTAGGGTCTAACTCACAACCCTAGTTTCTGTGCTGGCATTCTCCACCTGTTAATGTGTTTCGGGGAACTGTGTCAAGTTAGGGTGGCTTCCTGAAGGGTTGGA
            Consensus AAGTCACACAGCTGAGGTGCTGTAGGGCTAGGGTCTAACTCACAACCCTAGTTTCTGTGCTGGCATTCTCCACCTGTTAATGTGTTTCGGGGAACTGTGTCAAGTTAGGGTGGCTTCCTGAAGGGTTGGA

                     1171 1180      1190      1200      1210     1219
                     |--------+---------+---------+---------+--------|
           Ilvbl_JM8 GTTGGAGACTTGGTCATTTAGTGTCTCGTATTATGGTTTGTCCTCCCTG
    Ilvbl_refseq_3arm GTTGGAGACTTGGTCATTTAGTGTCTCGTATTATGGTTTGTCCTCCCTG
          Ilvbl_2_GR3 GTTGGAGACTTGGTCATTTAGTGTCTCGTATTATGGTTTGTCCTCCCTG
          Ilvbl_3_GR3 GTTGGAGACTTGGTCATTTAGTGTCTCGTATTATGGTTTGTCCTCCCTG
          Ilvbl_4_GR3 GTTGGAGACTTGGTCATTTAGTGTCTCGTATTATGGTTTGTCCTCCCTG
 targeting_vector_3ar GTTGGAGACTTGGTCATTTAGTGTCTCGTATTATGGTTTGTCCTCCCTG
            Consensus GTTGGAGACTTGGTCATTTAGTGTCTCGTATTATGGTTTGTCCTCCCTG
```

Ilvbl_JM8	direct sequencing JM8 cell line
Ilvbl_refseq_3arm	mouse reference sequence
targeting_vector_3ar	direct sequencing targeting vector arm
Ilvbl_2_GR3	direct sequencing genotyping PCR clone 2
Ilvbl_3_GR3	direct sequencing genotyping PCR clone 3
Ilvbl_4_GR3	direct sequencing genotyping PCR clone 4

Ppp4c 5' homology arm

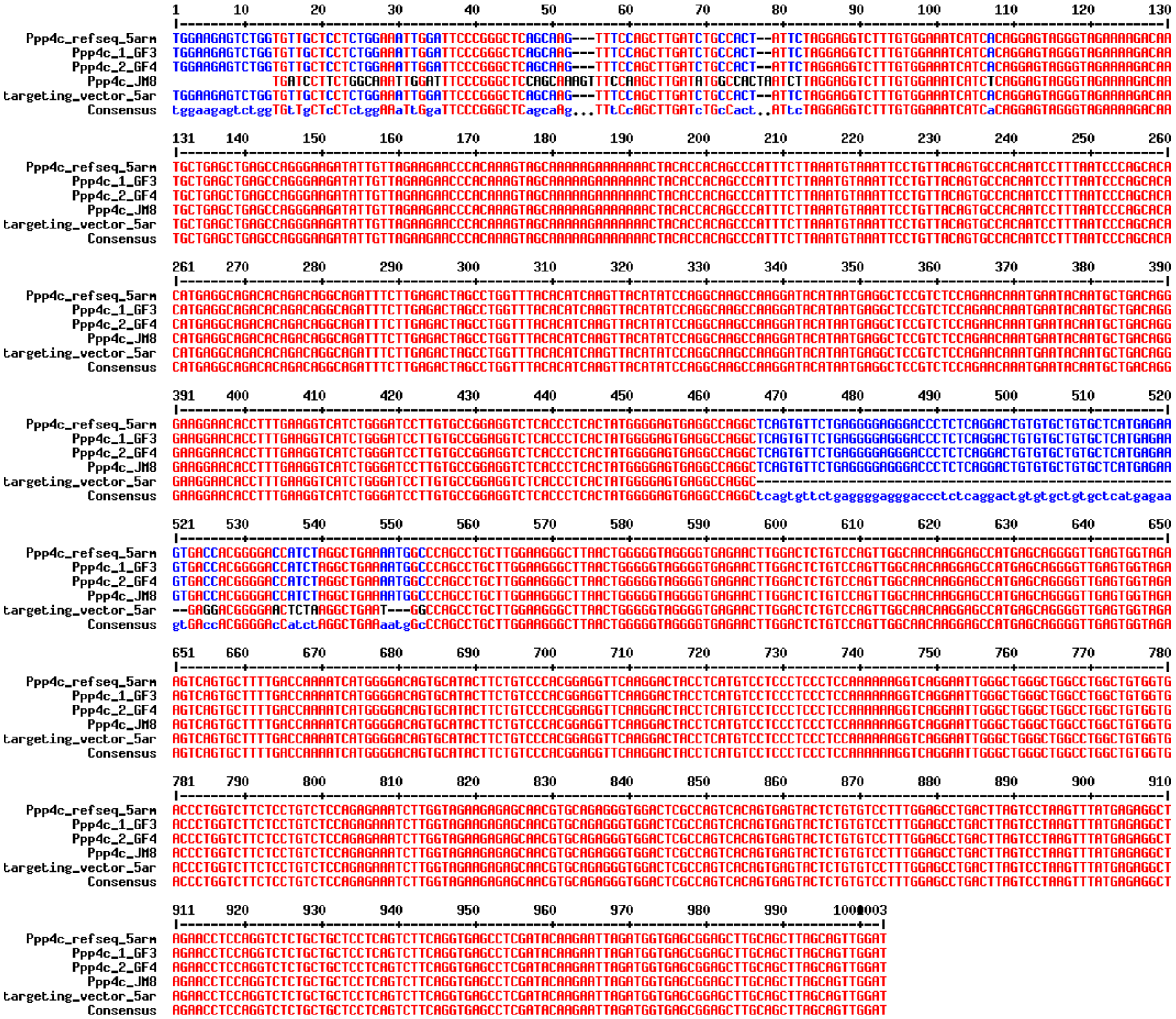

Ppp4c_JM8	direct sequencing JM8 cell line
Ppp4c_refseq_5arm	mouse reference sequence
targeting_vector_5ar	direct sequencing targeting vector arm
Ppp4c_1_GF3	direct sequencing genotyping PCR clone 1
Ppp4c_2_GF3	direct sequencing genotyping PCR clone 2

Ppp4c 3' homology arm

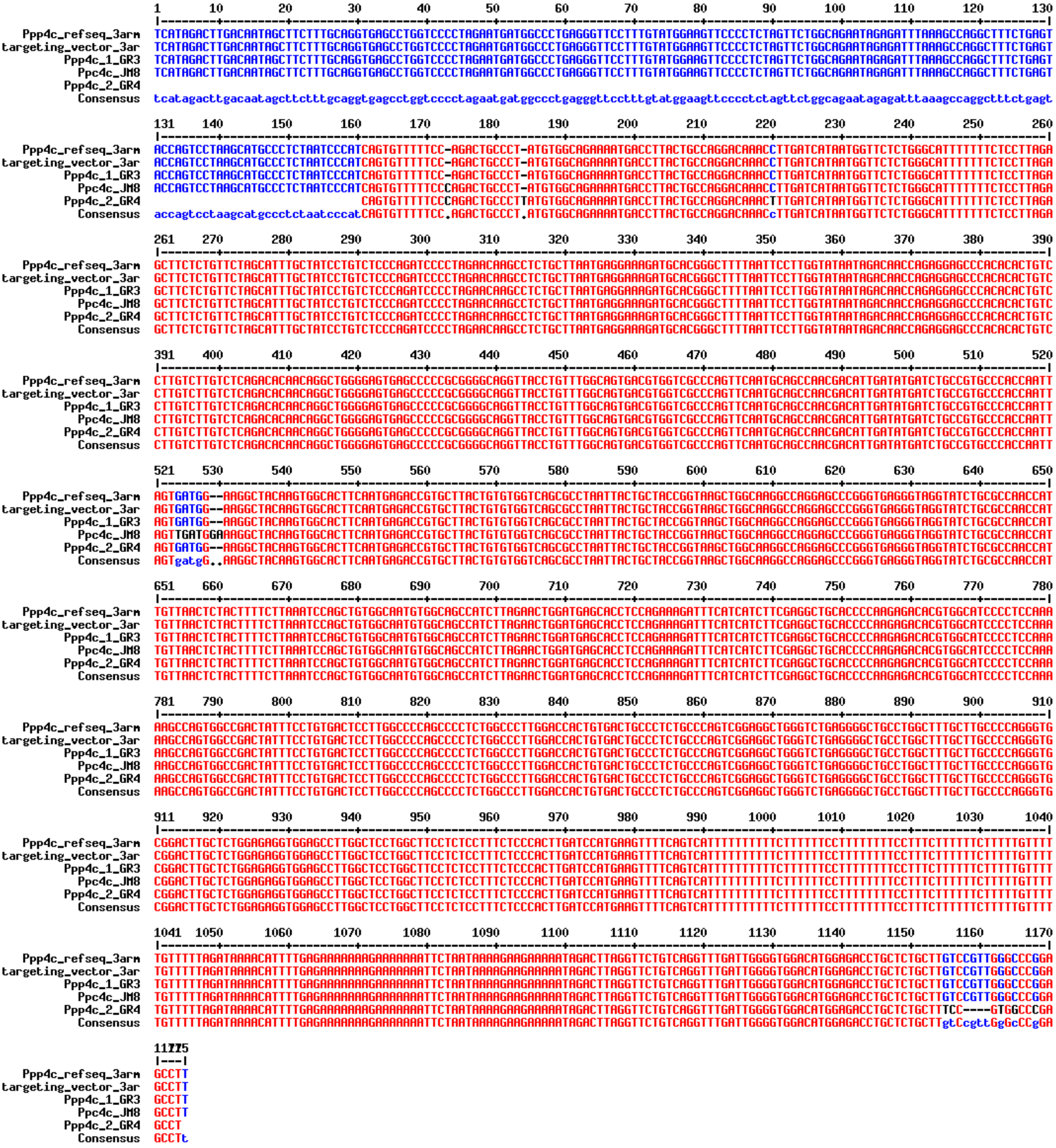

Ppp4c_JM8	direct sequencing JM8 cell line
Ppp4c_refseq_3arm	mouse reference sequence
targeting_vector_3ar	direct sequencing targeting vector arm
Ppp4c_1_GR3	direct sequencing genotyping PCR clone 1
Ppp4c_2_GR3	direct sequencing genotyping PCR clone 2

Ramp3 5' homology arm

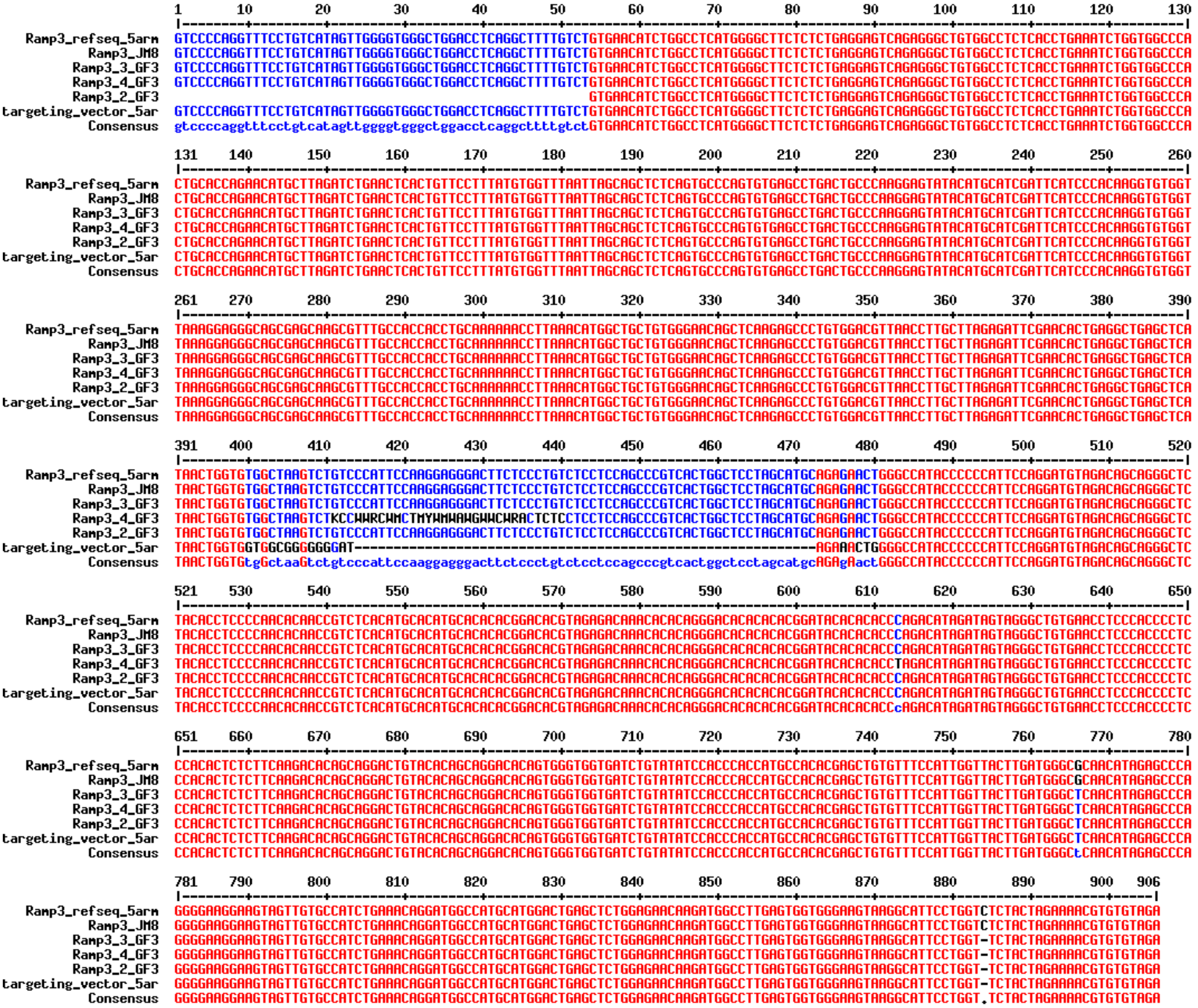

Ramp3_JM8	direct sequencing JM8 cell line
Ramp3_refseq_5arm	mouse reference sequence
targeting_vector_5ar	direct sequencing targeting vector arm
Ramp3_2_GF3	direct sequencing genotyping PCR clone 2
Ramp3_3_GF3	direct sequencing genotyping PCR clone 3
Ramp3_4_GF3	direct sequencing genotyping PCR clone 4

Ramp3 3' homology arm

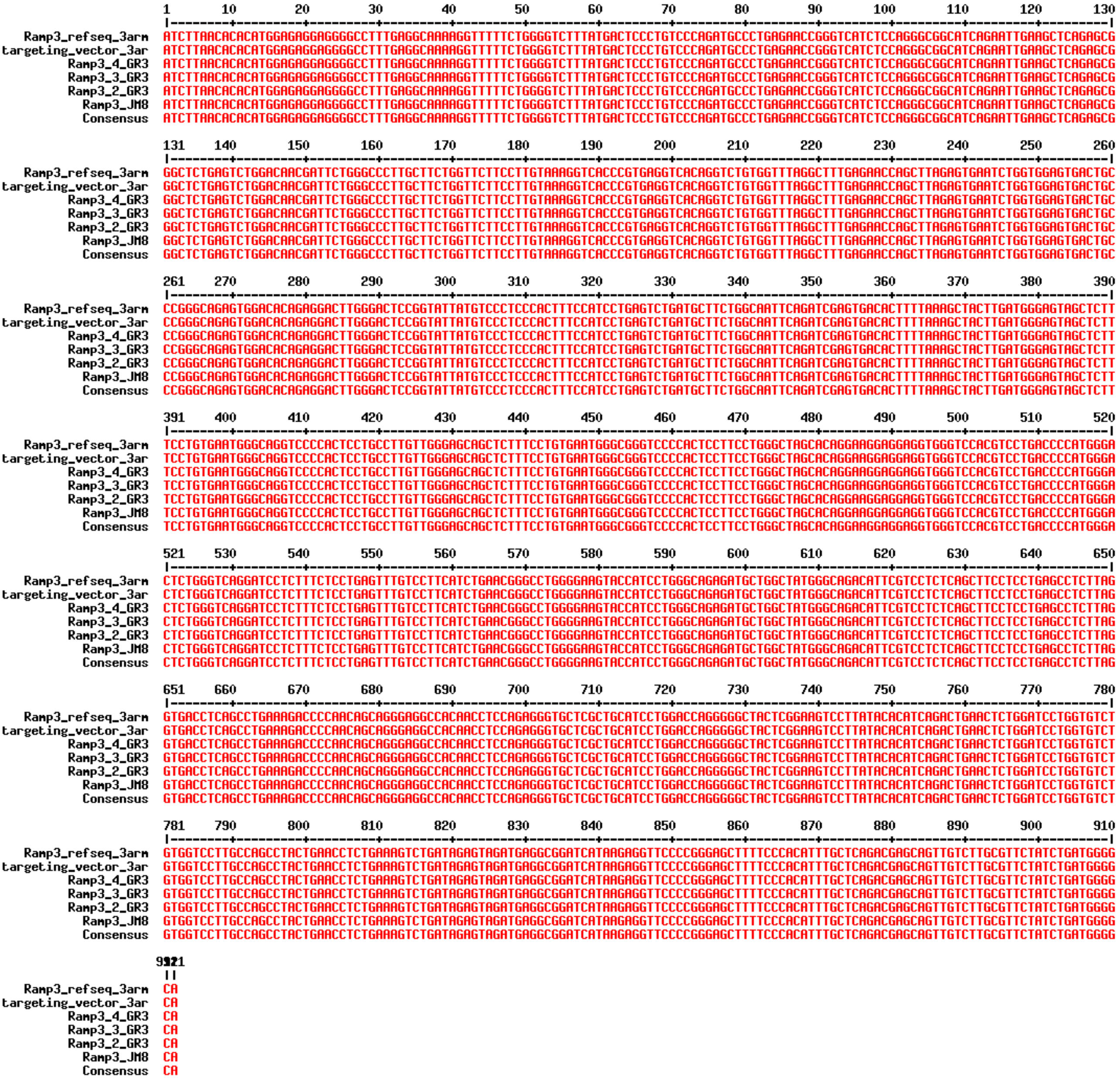

Ramp3_JM8	direct sequencing JM8 cell line
Ramp3_refseq_3arm	mouse reference sequence
targeting_vector_3ar	direct sequencing targeting vector arm
Ramp3_2_GR3	direct sequencing genotyping PCR clone 2
Ramp3_3_GR3	direct sequencing genotyping PCR clone 3
Ramp3_4_GR3	direct sequencing genotyping PCR clone 4

Eif4a3 5’ homology arm

```
                      1        10        20        30        40        50        60        70        80        90        100       110       120      130
                      |--------+---------+---------+---------+---------+---------+---------+---------+---------+---------+---------+---------+---------|
Eif4a3_refseq_5arm    GGAACAGAAAAAGAACCCCCTGTCATGTTTTAAACGCCTGGGTAAAGATTTTGAGATTCATTCCTAAATACCGCTGGAAGCCACTGCAGGGCTCTAAGCAAAATTGACAGGATCGGGCTTACAGTTTGAA
targeting_vector_5ar   GAACAGAAAAAGAACCCCCTGTCATGTTTTAAACGCCTGGGTAAAGATTTTGAGATTCATTCCTAAATACCGCTGGAAGCCACTGCAGGGCTCTAAGCAAAATTGACAGGATCGGGCTTACAGTTTGAA
Eif4a3_2_GF3          GGAACAGAAAAAGAACCCCCTGTCATGTTTTAAACGCCTGGGTAAAGATTTTGAGATTCATTCCTAAATACCGCTGGAAGCCACTGCAGGGCTCTAAGCAAAATTGACAGGATCGGGCTTACAGTTTGAA
Eif4a3_3_GF3          GGAACAGAAAAAGAACCCCCTGTCATGTTTTAAACGCCTGGGTAAAGATTTTGAGATTCATTCCTAAATACCGCTGGAAGCCACTGCAGGGCTCTAAGCAAAATTGACAGGATCGGGCTTACAGTTTGAA
Eif4a3_JM8
Consensus             ggaacagaaaaagaacccccctgtcatgttttaaacgcctgggtaaagattttgagattcattcctaaataccgctggaagccactgcagggctctaagcaaaattgacaggatcgggcttacagtttgaa

                      131      140       150       160       170       180       190       200       210       220       230       240       250      260
                      |--------+---------+---------+---------+---------+---------+---------+---------+---------+---------+---------+---------+---------|
Eif4a3_refseq_5arm    AGTTTATCTTGGTGTCTGGATTTGGAGTTAGAAGAGGAGGCGGGGGTGATGGCCGGACTGTAGAAGAGAGATGCTGATGTTGTCGTTGAGTTGGAGCTGGATTTTTTTTTTTTTTTTTTTTTTTTTGAGTG
targeting_vector_5ar  AGTTTATCTTGGTGTCTGGATTTGGAGTTAGAAGAGGAGGCGGGGGTGATGGCCGGACTGTAGAAGAGAGATGCTGATGTTGTCGTTGAGTTGGAGCTGGATTTTTTTTTTTTTTTTTTTTTTTTTGAGTG
Eif4a3_2_GF3          AGTTTATCTTGGTGTCTGGATTTGGAGTTAGAAGAGGAGGCGGGGGTGATGGCCGGACTGTAGAAGAGAGATGCTGATGTTGTCGTTGAGTTGGAGCTGGATTTTTTTTTTTTTTTTTTTTTT--GAGTG
Eif4a3_3_GF3          AGTTTATCTTGGTGTCTGGATTTGGAGTTAGAAGAGGAGGCGGGGGTGATGGCCGGACTGTAGAAGAGAGATGCTGATGTTGTCGTTGAGTTGGAGCTGGATTTTTTTTTTTTTTTTTTTTTTTTTGAGTG
Eif4a3_JM8                                                                                                                     TTTTTTTTTTTTTTTTTTTTGAGTG
Consensus             agtttatcttggtgtctggatttggagttagaagaggaggcgggggtgatggccggactgtagaagagagatgctgatgttgtcgttgagttggagctggattttTTTTTTTTTTTTTTTTTTttGAGTG

                      261      270       280       290       300       310       320       330       340       350       360       370       380      390
                      |--------+---------+---------+---------+---------+---------+---------+---------+---------+---------+---------+---------+---------|
Eif4a3_refseq_5arm    AATGGTCGGTCATGCTGCTGGTGTGTGTTTAGGAGCATTTAAGGGAGGCCAAATGAGCTTTGTCTTTTTGTTTCTGTCCTTTTCTTTTTTCCTTTTTTTTTTTGAGACTTTCTCTTTGGAGCCCTGGCCG
targeting_vector_5ar  AATGGTCGGTCATGCTGCTGGTGTGTGTTTAGGAGCATTTAAGGGAGGCCAAATGAGCTTTGTCTTTTTGTTTCTGTCCTTTTCTTTTTTCCTTTTTTTTTTTGAGACTTTCTCTTTGGAGCCCTGGCCG
Eif4a3_2_GF3          AATGGTCGGTCATGCTGCTGGTGTGTGTTTAGGAGCATTTAAGGGAGGCCAAATGAGCTTTGKCTTTTTGTTTCTGKCCTTTTCTTTTTTCCTTTTTTTTTTTGAGACTTTCTCTTTGGAGCCCTGGCCG
Eif4a3_3_GF3          AATGGTCGGTCATGCTGCTGGTGTGTGTTTAGGAGCATTTAAGGGAGGCCAAATGAGCTTTGTCTTTTTGTTTCTGTCCTTTTCTTTTTTCCTTTTTTTTTTTGAGACTTTCTCTTTGGAGCCCTGGCCG
Eif4a3_JM8            AATGGTCGGTCATGCTGCTGGTGTGTGTTTAGGAGCATTTAAGGGAGGCCAAATGAGCTTTGTCTTTTTGTTTCTGTCCTTTTCTTTTTTCCTTTTTTTTTTTGAGACTTTCTCTTTGGAGCCCTGGCCG
Consensus             AATGGTCGGTCATGCTGCTGGTGTGTGTTTAGGAGCATTTAAGGGAGGCCAAATGAGCTTTGtCTTTTTGTTTCTGtCCTTTTCTTTTTTCCTTTTTTTTTTTGAGACTTTCTCTTTGGAGCCCTGGCCG

                      391      400       410       420       430       440       450       460       470       480       490       500       510      520
                      |--------+---------+---------+---------+---------+---------+---------+---------+---------+---------+---------+---------+---------|
Eif4a3_refseq_5arm    TCCTGGAATTCTTTGTAGACCAGGCTGGCCTCAAACTCAGAGATTCCCTGCCTCTGCCTCCCAGATGGTGGGGTTAAA-GGTGTGCCACACCAAGCC-AGGATTGTTGTTGTTGTTGTTGTTTTGAGGTA
targeting_vector_5ar  TCCTGGAATTCTTTGTAGACCAGGCTGGCCTCAAACTCAGAGATTCCCTGCCTCTGCCTCCCAGATGGTGGGGTTAAA-GGTGTGCCACACCAAGCC-AGGATTGTTGTTGTTGTTGTTGTTTTGAGGTA
Eif4a3_2_GF3          TCCTGGAATTCTTTGTAGACCAGGCTGGCCTCAAACTCAGAGATTCCCTGCCTCTGCCTCCCAGATGGTGGGGTTAAA-GGTGTGCCACACCAAGCC-AGGATTGTTGTTGTTGTTGTTGTTTTGAGGTA
Eif4a3_3_GF3          TCCTGGAATTCTTTGTAGACCAGGCTGGCCTCAAACTCAGAGATACCCTGCCTCTGCCTCCCAGATGRKGGGGWTAMWAGGWGWRMCACACCAAGCYYASSAWWGTKGTWGWTGYTGTYGTKTWGAGGTA
Eif4a3_JM8            TCCTGGAATTCTTTGTAGACCAGGCTGGCCTCAAACTCAGAGATTCCCTGCCTCTGCCTCCCAGATGGTGGGGTTAAA-GGTGTGCCACACCAAGCC-AGGATTGTTGTTGTTGTTGTTGTTTTGAGGTA
Consensus             TCCTGGAATTCTTTGTAGACCAGGCTGGCCTCAAACTCAGAGATtCCCTGCCTCTGCCTCCCAGATGGgtGGGGtTAaa.GGtGtgcCACACCAAGCc.AggAttGTtGTtGtTGtTGTtGTtTtGAGGTA

                      521      530       540       550       560       570       580       590       600       610       620       630       640      650
                      |--------+---------+---------+---------+---------+---------+---------+---------+---------+---------+---------+---------+---------|
Eif4a3_refseq_5arm    GGGTCTCTACCTAGCCCTAGCTGCCCTGGAACTCACTAGACCAGGAAGATTGGTCCCTCCCATTTTTAATGATACCCAGCTTGGGTTTTCGGTAATCCCAGATGTAGGCAAGTTGGCAGCCAAGAATAGC
targeting_vector_5ar  GGGTCTCTACCTAGCCCTAGCTGCCCTGGAACTCACTAGACCAGGAAGATTGCTCCCTCCCATTTTTAATGATACCCAGCTTGGGTTTTCGGTAATCCCAGATGTAGGCAAGTTGGCAGCCAAGAATAGC
Eif4a3_2_GF3          GGGTCTCTACCTAGCCCTAGCTGCCCTGGAACTCACTAGACCAGGAAGATTGGTCCCTCCCATTTTTAATGATACCCAGCTTGGGTTTTCGGTAATCCCAGATGTAGGCAAGTTGGCAGCCAAGAATAGC
Eif4a3_3_GF3          GRGTCTCWASSTAGCCC-AGCWGMCCKGGARCKCRCTAGACCAGGAAGATYGGTCCSTCYCATTTTTAAYSATWCSCAGCTYGGGTTTTCGGTAATSMCAGAYGTAGKCAAGTTGGCAGCCAAGAAKAGC
Eif4a3_JM8            GGGTCTCTACCTAGCCCTAGCTGCCCTGGAACTCACTAGACCAGGAAGATTGGTCCCTCCCATTTTTAATGATACCCAGCTTGGGTTTTCGGTAATCCCAGATGTAGGCAAGTTGGCAGCCAAGAATAGC
Consensus             GgGTCTCtAccTAGCCCtAGCtGcCCtGGAaCtCaCTAGACCAGGAAGATtGgTCCcTCcCATTTTTAAtgATaCcCAGCTtGGGTTTTCGGTAATccCAGAtGTAGgCAAGTTGGCAGCCAAGAAtAGC

                      651      660       670       680       690       700       710       720       730       740       750       760       770      780
                      |--------+---------+---------+---------+---------+---------+---------+---------+---------+---------+---------+---------+---------|
Eif4a3_refseq_5arm    ATTACAAGTCCTGTGTTGAGCTGGCAAGTGTCACTAGCTACATCTTAAAAGAGTGTAAAAATAAACTACAACAAGCCAGTCTCACAGCTTTAATCCCAGCACTTGGGAGACAGAGGTCAGTCTACAAAGT
targeting_vector_5ar  ATTACAAGTCCTGTGTTGAGCTGGCAAGTGTCACTAGCTACATCTTAAAAGAGTGTAAAAATAAACTACAACAAGCCAGTCTCACAGCTTTAATCCCAGCACTTGGGAGACAGAGGTCAGTCTACAAAGT
Eif4a3_2_GF3          ATTACAAGTCCTGTGTTGAGCTGGCAAGTGTCACTAGCTACATCTTAAAAGAGTGTAAAAATAAACTACAACAAGCCAGTCTCACAGCTTTAATCCCAGCACTTGGGAGACAGAGGTCAGTCTACAAAGT
Eif4a3_3_GF3          ATTACAAGTCMTGTGYTGAGCTGGCAAGTGTCWCTAGCTACATCTGAAAAGAGTGTAAAAATAAACTACAACAAGCCAGTCTCACAGCTTTAATCYCAGCACTWGGGAGACAGAGGTCAGTCTACAAAGT
Eif4a3_JM8            ATTACAAGTCCTGTGTTGAGCTGGCAAGTGTCACTAGCTACATCTTAAAAGAGTGTAAAAATAAACTACAACAAGCCAGTCTCACAGCTTTAATCCCAGCACTTGGGAGACAGAGGTCAGTCTACAAAGT
Consensus             ATTACAAGTCcTGTGtTGAGCTGGCAAGTGTCaCTAGCTACATCTtAAAAGAGTGTAAAAATAAACTACAACAAGCCAGTCTCACAGCTTTAATCcCAGCACTtGGGAGACAGAGGTCAGTCTACAAAGT

                      781      790       800       810       820       830       840       850       860       870       880       890       900      910
                      |--------+---------+---------+---------+---------+---------+---------+---------+---------+---------+---------+---------+---------|
Eif4a3_refseq_5arm    GAGTTCAAGATAGCCAGGGCTCTGTTACTTAGAGAAACCCTATCTTGAACAACCCCTCACCACCCCCCCACCACCCCCACACACACCAAAGCAAACCAACAACATCTTTTCTGTATAAATTATGTCTGCT
targeting_vector_5ar  GAGTTCAAGATAGCCAGGGCTCTGTTACTTAGAGAAACCCTATCTTGAACAACCCCTCACCACCCCCCCACCACCCCCACACACACCAAAGCAAACCAACAACATCTTTTCTGTATAAATTATGTCTGCT
Eif4a3_2_GF3          GAGTTCAAGATAGCCAGGGCTCTGTTACTTAGAGAAACCCTATCTTGAACAACCCCTCACCACCCCCCCACCACCCCCACACACACCAAAGCAAACCAACAACATCTTTTCTGTATAAATTATGTCTGCT
Eif4a3_3_GF3          GAGTTCAAGATAGCCAGGGCTSTGTKACTTAGAGAARCCCTATCTTGAACAASSCCTCRSCASMSCCCCMCCWMCSCCACACACMYCAAAGCAARCCAACAACATCTTTTCWGTATAAATTAYGTCTGCT
Eif4a3_JM8            GAGTTCAAGATAGCCAGGGCTCTGTTACTTAGAGAAACCCTATCTTGAACAACCCCTCACCACCCCCCCACCACCCCCACACACACCAAAGCAAACCAACAACATCTTTTCTGTATAAATTATGTCTGCT
Consensus             GAGTTCAAGATAGCCAGGGCTcTGTtACTTAGAGAAaCCCTATCTTGAACAAccCCTCacCAcccCCCCaCCacCcCCACACACacCAAAGCAAaCCAACAACATCTTTTCtGTATAAATTAtGTCTGCT

                      911      920       930       940       950       960       970       980      991
                      |--------+---------+---------+---------+---------+---------+---------+--------+|
Eif4a3_refseq_5arm    TATTTGGAAAGTACTTGTGTTTTTGTTGTTGTTTGTTTTGAGACAGGTCTCTACATAGTCTTGTCTGTCCTGGAACACTCT
targeting_vector_5ar  TATTTGGAAAGTACTTGTGTTTTTGTTGTTGTTTGTTTTGAGACAGGTCTCTACATAGTCTTGTCTGTCCTGGAACACTCT
Eif4a3_2_GF3          TATTTGGAAAGTACTTGTGTTTTTGTTGTTGTTTGTTTTGAGACAGGTCTCTACATAGTCTTGTCTGTCCTGGAACACTCT
Eif4a3_3_GF3          TATTTGGAAAGTACTTGTGTTTTTGTTGTTGTTTGTTTTGAGACAGGTCTCWACATAGTCTTGTCTGTCCTGGAACACTMT
Eif4a3_JM8            TATTTGGAAAGTACTTGTGTTTTTGTTGTTGTTTGTTTTGAGACAGGTCTCTACATAGTCTTGTCTGTCCTGGAACACTCT
Consensus             TATTTGGAAAGTACTTGTGTTTTTGTTGTTGTTTGTTTTGAGACAGGTCTCtACATAGTCTTGTCTGTCCTGGAACACTcT
```

Eif4a3_JM8	direct sequencing JM8 cell line
Eif4a3_refseq_5arm	mouse reference sequence
targeting_vector_5ar	direct sequencing targeting vector arm
Eif4a3_2_GF3	direct sequencing genotyping PCR clone 2
Eif4a3_3_GF3	direct sequencing genotyping PCR clone 3

Eif4a3 3' homology arm

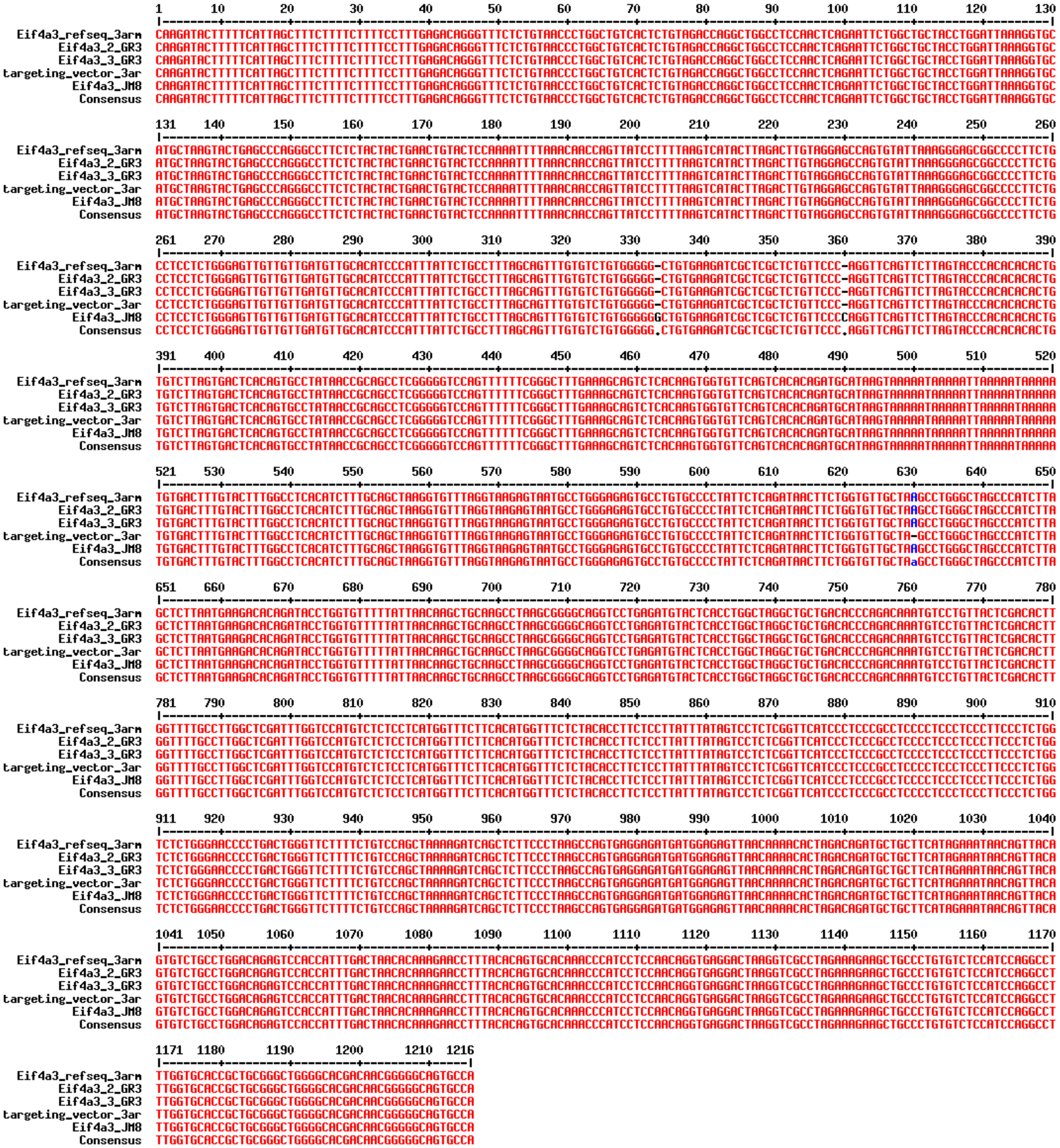

Eif4a3_JM8	direct sequencing JM8 cell line
Eif4a3_refseq_3arm	mouse reference sequence
targeting_vector_3ar	direct sequencing targeting vector arm
Eif4a3_2_GR3	direct sequencing genotyping PCR clone 2
Eif4a3_3_GR3	direct sequencing genotyping PCR clone 3
